# Characterization of *Campylobacter jejuni* proteome profiles in co-incubation scenarios

**DOI:** 10.3389/fmicb.2023.1247211

**Published:** 2023-11-09

**Authors:** Annika Dreyer, Christof Lenz, Uwe Groß, Wolfgang Bohne, Andreas Erich Zautner

**Affiliations:** ^1^Institute for Medical Microbiology and Virology, University Medical Center Göttingen, Göttingen, Germany; ^2^Bioanalytical Mass Spectrometry Group, Max Planck Institute for Multidisciplinary Sciences, Göttingen, Germany; ^3^Department of Clinical Chemistry, University Medical Center Göttingen, Göttingen, Germany; ^4^Institute of Medical Microbiology and Hospital Hygiene, Medical Faculty, Otto-von-Guericke University Magdeburg, Magdeburg, Germany; ^5^Center for Health and Medical Prevention (CHaMP), Otto-von-Guericke University Magdeburg, Magdeburg, Germany

**Keywords:** *Campylobacter jejuni*, co-incubation, *Enterococcus faecalis*, *Enterococcus faecium*, *Staphylococcus aureus*, bile acids, proteomics

## Abstract

In dynamic microbial ecosystems, bacterial communication is a relevant mechanism for interactions between different microbial species. When *C. jejuni* resides in the intestine of either avian or human hosts, it is exposed to diverse bacteria from the microbiome. This study aimed to reveal the influence of co-incubation with *Enterococcus faecalis*, *Enterococcus faecium*, or *Staphylococcus aureus* on the proteome of *C. jejuni* 81–176 using data-independent-acquisition mass spectrometry (DIA-MS). We compared the proteome profiles during co-incubation with the proteome profile in response to the bile acid deoxycholate (DCA) and investigated the impact of DCA on proteomic changes during co-incubation, as *C. jejuni* is exposed to both factors during colonization. We identified 1,375 proteins by DIA-MS, which is notably high, approaching the theoretical maximum of 1,645 proteins. *S. aureus* had the highest impact on the proteome of *C. jejuni* with 215 up-regulated and 230 down-regulated proteins. However, these numbers are still markedly lower than the 526 up-regulated and 516 down-regulated proteins during DCA exposure. We identified a subset of 54 significantly differentially expressed proteins that are shared after co-incubation with all three microbial species. These proteins were indicative of a common co-incubation response of *C. jejuni*. This common proteomic response partly overlapped with the DCA response; however, several proteins were specific to the co-incubation response. In the co-incubation experiment, we identified three membrane-interactive proteins among the top 20 up-regulated proteins. This finding suggests that the presence of other bacteria may contribute to increased adherence, e.g., to other bacteria but eventually also epithelial cells or abiotic surfaces. Furthermore, a conjugative transfer regulon protein was typically up-expressed during co-incubation. Exposure to both, co-incubation and DCA, demonstrated that the two stressors influenced each other, resulting in a unique synergistic proteomic response that differed from the response to each stimulus alone. Data are available via ProteomeXchange with identifier PXD046477.

## 1. Introduction

*Campylobacter jejuni* belongs to the most frequently diagnosed bacterial gastrointestinal pathogens in humans worldwide (Acheson and Allos, 2001). In the developed world, foodborne infections most commonly occur after consumption of cross-contaminated food, prepared in parallel with poultry meat., whereas *Campylobacter* spp. belong to the natural commensal microbiome in poultry (Skirrow, 1991). Additional sources for infections are water, raw milk or other livestock animals (Blaser et al., 1980, 1983; Szewzyk et al., 2000). Symptoms of campylobacteriosis include severe bloody diarrhea, fever, abdominal cramps and nausea. Furthermore, *Campylobacter* infections are associated with severe follow-up diseases, for example the Guillain-Barré syndrome, a neural disease that can lead to paralyzes and damage of the nervous system (Rees and Hughes, 1995; Sejvar et al., 2011).

The ideal growth temperature for the Gram-negative, helical-shaped and microaerophilic bacterium lies between 37°C and 42°C. Due to its broad spectrum of virulence-associated factors that enable the survival in varying environmental conditions, *C. jejuni* can successfully colonize the gut of avian and mammal hosts. One of these virulence-associated factors is the ability to survive high concentrations of bile acid in the human or animal gut. Among the diverse functions of bile is the solubilization and emulsification of fat, which makes it an important biological detergent (Begley et al., 2005; Chiang, 2017). Under the exposition of bile acids, the composition of fatty acids and phospholipids of the bacterial cell membranes are altered, which leads to instabilities in the cell’s surface and consequently to the disruption of the cell (Taranto et al., 2003). Furthermore, DNA damages might be induced by the presence of bile acid in different bacteria, such as *E. coli* (Kandell and Bernstein, 1991; Begley et al., 2005). To overcome this stress, bacterial gut inhabitants have developed several mechanisms to cope with bile acid and are able to tolerate varying concentrations of bile.

Co-incubation can have several important positive or negative effects on the growth of different bacteria. In presence of other microbes, some pathogenic bacteria show an increase in in their virulence (Stacy et al., 2016; Fast et al., 2018). However, proteomic studies on co-incubation remain rare. A proteomic study by García-Pérez and coworkers has shown that co-incubation can reduce the number of extracellular proteins in microbial communities in wounds (García-Pérez et al., 2018). In addition, co-incubation of different bacteria with yeasts, such as *C. albicans,* has shown positive effects on the growth of both species, probably due to the release of nutrients into the medium or beneficial changes in pH (Ellepola et al., 2019). During co-incubation with other bacteria, *C. jejuni* has been shown to interact with a variety of other bacteria, for instance *Bifidobacterium longum* which prevents the adherence of *C. jejuni* to intestinal tract cells (Quinn et al., 2020a,b). A combination of different bacteria that include *E. faecium* can lead to a decrease of *C. jejuni* in the gastro-intestinal tract of poultry (Neveling and Dicks, 2021). Anis and colleagues showed that studying the co-incubation of *C. jejuni* with other bacteria might be an interesting topic, as the bacterial interaction might enhance *C. jejuni* survival when exposed to external stresses, such as the presence of oxygen (Anis et al., 2022). Further studies about co-cultivation of *C. jejuni* with *E. coli* and *L. monocytogenes,* showed that the adhesion potential of *C. jejuni* to all tested surfaces was significantly increased. In summary, this study suggests that the presence of other (foodborne) bacteria may increase the adhesion of *C. jejuni*, and thus, co-incubation might contribute to its pathogenicity (Klančnik et al., 2020). The only co-cultivation study that investigated transcriptomics or proteomics in *C. jejuni* during co-cultivation involved eukaryotic cells, specifically human INT 407 and Caco-2 epithelial cells (Negretti et al., 2019). Thus, our study aiming the proteomic adaptations of *C. jejuni* to bacterial co-cultivation is novel and covers a so far unexplored subject.

To our knowledge. Proteomic or transcriptomic studies of *C. jejuni* with other bacteria do not exist so far. The only co-cultivation studies that investigated transcriptomics or proteomics in *C. jejuni* during co-cultivation include eukaryotic cells, such as human INT 407 and Caco-2 epithelial cells (Negretti et al., 2019). Thus, our study aiming the proteomic adaptations of *C. jejuni* to bacterial co-cultivation is novel and covers a so far unexplored subject.

In this study, we aimed to observe the impact of co-incubation on the *C. jejuni* proteome and the possible effects of co-incubation on the bile acid response of the bacterium. Therefore, we analyzed the proteome of *C. jejuni* in co-incubation and under deoxycholate (DCA = deoxycholic acid) stress. DCA is a secondary bile acid, which is a product of dehydroxylation by gut microbiota and has been shown to have inhibiting effects on the growth of *C. jejuni* and other bacteria at a certain concentration (Lertpiriyapong et al., 2012; Vidal et al., 2021) and furthermore substantial effects on the proteome (Masanta et al., 2019). The three bacterial species chosen for co-incubation were less resistant toward DCA than *C. jejuni.*

One of the bacterial species chosen for the co-incubation study was *E. faecalis,* a Gram-positive, facultative anaerobic coccal opportunistic pathogen that belongs to the human commensal microbiome, but can also be found in environmental samples (Lebreton et al., 2014; Van Tyne and Gilmore, 2014; Fiore et al., 2019). Furthermore, we tested a close relative of *E. faecalis, E. faecium,* which is also an opportunistic pathogen of global importance due to its high antibiotic resistance potential (Lopes et al., 2006; Gorrie et al., 2019). The third bacterium used in this study was *Staphylococcus aureus,* another Gram-positive pathogen of high clinical relevance due to the high number of severe infections caused by multidrug resistant *S. aureus* (Klevens et al., 2007; Rasigade et al., 2014; Cheung et al., 2021).

This study aims to provide a deeper look at the co-incubation proteome of the pathogen *C. jejuni* with other bacteria that are usually present in the human body and the respective proteomic changes in presence of DCA. We used data-independent acquisition mass spectrometry (DIA-MS) to systematically compare the proteomic changes in co-incubation of the different bacteria with *C. jejuni* as well as the proteomic response to bile acid (DCA). This technique enables the quantitative analysis of every detectable compound in a sample of proteins and thus provides high reliability in the quantitative results (Huang et al., 2015). To our knowledge, this is the first proteomic co-incubation study on *C. jejuni*.

## 2. Materials and methods

### 2.1. Bacterial strains and growth conditions

*Campylobacter jejuni* 81–176 (purchased from the American Type Culture Collection: ATCC-BAA-2151) was used for all described experiments. *C. jejuni* was grown overnight on CAM-agar plates from Biomérieux (Marcy-l’Étoile, France) at 42°C. Mueller-Hinton (MH) broth served as liquid medium at 37°C. To generate a microaerophilic environment, the Gas Pak™ EZ Campy Container System by BD (Franklin Lakes, NJ, USA) and an anaerobic jar for incubation were used.

*Enterococcus faecalis* ATCC 700802 (V583; purchased from the American Type Culture Collection), *Enterococcus faecium* TX0016 (also purchased from the American Type Culture Collection: ATCC BAA-472) and *Staphylococcus aureus* NCTC 8325 (PS 47, purchased from the BCCM/LMG: LMG 21764) were used for co-incubation experiments and grown overnight on Columbia agar plates supplemented with sheep blood purchased from Biomérieux (Marcy-l’Étoile, France).

### 2.2. Co-incubation

For co-incubation experiments, the optical density at 600 nm (OD_600_) of *C. jejuni* was set to 0.5 and the OD_600_ of the respective other bacterium was set to 0.1. Incubation was performed in phosphate buffered saline (PBS) to avoid effects of the medium on the bile acid resistance. DCA was added to the medium at a concentration of 0.1% for *E. faecalis* and *E. faecium* and 0.075 for *S. aureus.* These concentrations are lethal to these Gram-positive bacteria when cultured individually. Incubation was carried out for 3 h at 37°C and shaking at 150 rpm. After three hours, a spot assay on Mueller-Hinton agar plates was done to show the survival of the bacteria after 3 h in a dilution series. Subsequently, protein extraction was done.

The three Gram-positive bacterial species without presence of *C. jejuni* served as positive control while the approaches of the Gram-positive bacteria with the respective amount of DCA served as negative control. All samples were prepared in biological triplicates.

### 2.3. Protein extraction from pellet

Cultures were centrifuged at 4,000 rpm for 10 min at 4°C. For protein-extraction from the pellet, the supernatant was discarded. For samples containing *C. jejuni,* pellets were resuspended in 2 mL 0.9% saline and kept on ice over the procedure. Subsequently, the Gram-negative cells were disrupted via sonification using a Branson sonifier 250 from Branson ultrasonics (Brookfield, Connecticut, USA) with the following settings: output control = 3, duty cycler = 30%. The sonification process was performed five times for 30 s followed by 30 s of cooling to avoid overheating of the proteins. Afterwards, the Gram-positive cells were disrupted using 0.75 g of 4 mm glass beads that were added to the samples and were subsequently treated in a “Fast prep 96 Homogenizer” (MP Biomedicals Germany GmbH, Eschwege, Germany) for 2 × 20 s, followed by centrifugation at 5,500 g for one minute. The supernatant was then removed and samples were centrifuged at 13,500 xg for 10 min at 4°C in a tabletop centrifuge. Finally, the supernatant was used for a Pierce assay, that was performed to determine the protein concentration of all samples. After this, the concentrations were adjusted to 1 μg/μL for DIA-MS analysis. For all samples, biological triplicates were prepared.

### 2.4. Data-independent-acquisition mass spectrometry (DIA-MS)

Protein samples were loaded onto a 4–12% NuPAGE Novex Bis-Tris Minigels (Invitrogen) and run into the gel for 1.5 cm. Following Coomassie staining, the protein areas were cut out, diced, and subjected to reduction with dithiothreitol, alkylation with iodoacetamide and finally overnight digestion with trypsin was performed. Tryptic peptides were extracted from the gel, the solution dried in a Speedvac and kept at −20°C for further analysis.

Protein digests were analyzed on a nanoflow chromatography system (nanoElute) hyphenated to a hybrid timed ion mobilityquadrupole-time of flight mass spectrometer (timsTOF Pro, all Bruker Daltonics GmbH & Co. KG, Bremen, Germany). In brief, 250 ng equivalents of peptides were dissolved in loading buffer (2% acetonitrile, 0.1% trifluoroacetic acid in water), enriched on a reversed-phase C18 trapping column (0.3 cm × 300 μm, Thermo Fisher Scientific) and separated on a reversed phase C18 column with an integrated CaptiveSpray Emitter (Aurora 25 cm × 75 μm, IonOpticks) using a 50 min linear gradient of 5–35% acetonitrile / 0.1% formic acid (v:v) at 250 nL min^−1^, and a column temperature of 50°C. For identification, representative samples were analyzed in PASEF acquisition mode using default manufacturer’s settings [n = 12] (Meier et al., 2018). For identification and quantification samples were analyzed in diaPASEF mode using a customized 16×2 window acquisition scheme (Skowronek et al., 2022). For each biological replicate, three technical replicates were performed in diaPASEF mode for quantitation.

### 2.5. Data processing and statistics

The data processing was performed using the Spectronaut v16.0.220606.53000 software package (Biognosys AG, Schlieren, Switzerland). Identification of proteins as well as hybrid spectral library generation from 12×2 DDA acquisitions and 12×2 DIA acquisitions experiments were done using the Pulsar search engine against UniProtKB *C. jejuni* 81–176, *E. faecalis* ATCC 700802, *E. faecium* TX0016 and *S. aureus* NCTC 8325 proteomes using the default parameters. The False Discovery Rate (FDR) was set to 1% on the spectral, peptide and protein group levels for all samples. DIA quantification was performed with up to 6 fragments per peptide and up to 10 peptides per protein. A dynamic retention time alignment was done, as well as dynamic mass recalibration and quartile normalization, for the 1% FDR. Imputation of global data was executed for the final results table.

Perseus v1.6.2.2 was used for the statistical analysis and for generation of volcano plots to compare the different samples (Storey and Tibshirani, 2003; Tyanova et al., 2016). As significant regulation level, two-fold up- or down-expression was chosen. Proteins present in 80% of the samples were considered for further analysis. For volcano-plot generation in Perseus, a t-test was chosen with a number of randomizations = 250 and a FDR of 0.05 (Storey and Tibshirani, 2003). All proteins that are described in the following as up- or down-expressed were significantly regulated, if not otherwise stated.

Clusters of orthologous genes (COG)-categories were assigned to the proteins using the online-tool eggNOGmapper v 2.18 (Huerta-Cepas et al., 2017, 2019; Cantalapiedra et al., 2021). To identify commonly expressed proteins, Venn diagrams were generated utilizing InteractiVenn (Heberle et al., 2015). All Plots were generated using matplotlib in python3 (Van Rossum and Drake, 2009).

## 3. Results and discussion

### 3.1. Identification of *Campylobacter jejuni* proteins that are commonly regulated during co-incubation with different Gram-positive bacteria

The interbacterial communication between *C. jejuni* and other bacterial species remains poorly explored to date, lacking comprehensive investigation. Our research is aimed to investigate mechanisms of this cross-talk and its potential implications in various ecological and pathogenic contexts.

**Figure 1:**
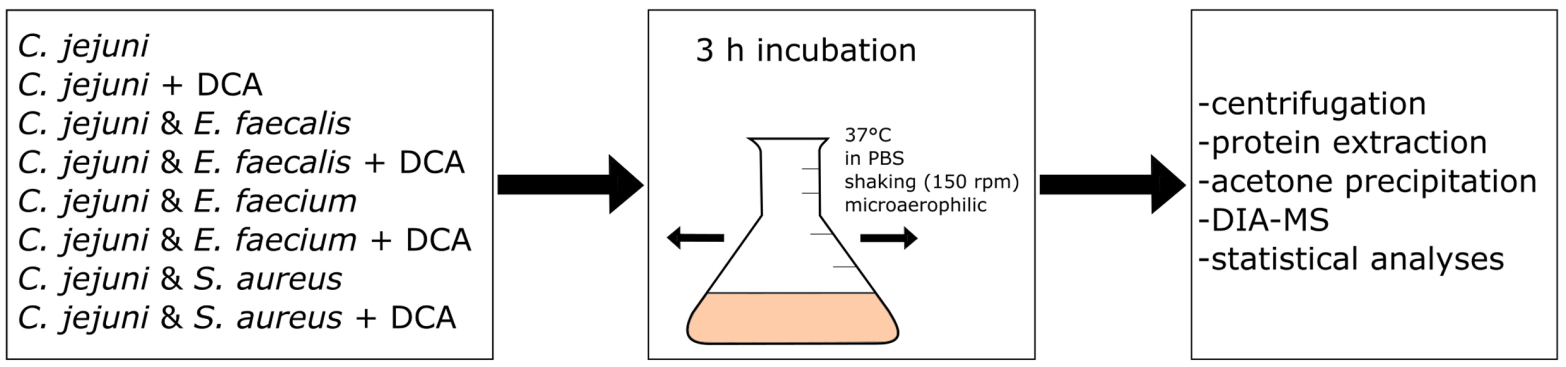
Workflow scheme: eight different approaches of mono- or co-incubation were prepared and incubated at 150 rpm and 37°C for three hours. After incubation, the approaches were centrifuged and the proteins were extracted and precipitated with acetone. Data-Independent Acquisition Mass Spectrometry (DIA-MS) was performed, followed by data analysis and statistical analysis.

We hypothesized that co-incubation of *C. jejuni* with other bacterial species triggers a proteomic response in *C. jejuni*. Three different Gram-positive species were chosen for co-incubation with *C. jejuni*, namely *E. faecalis*, *E. faecium* and *S. aureus*, which are all putative inhabitants of the intestinal host microbiome. The bacteria were incubated for three hours at 37°C in PBS, without nutrient supply since we were not interested in responses due to different degrees of nutrient competition (see scheme of the workflow in Figure 1). Instead, we aimed to target responses resulting from direct bacterial contact or from interactions with secreted molecules. Using volcano-plots generated from DIA-MS data, we compared the proteome of *C. jejuni* in monoculture with each of the three bacteria with *C. jejuni* in co-incubations.

Co-incubation resulted in all cases in an altered proteomic profile, whose dimension depends on the species used for co-incubation. With *S. aureus,* the changes in the proteomic profile exhibited the highest intensity with 445 differentially expressed proteins.

It is well known that *S. aureus* produces several toxins and hemolysins that might act against other bacteria (Shinefield, 1963; Otto, 2014). Conversely, *S. aureus* can also secrete beneficial substances for other microorganisms and co-exist in polymicrobial communities, which can be advantageous for infections (Nguyen and Oglesby-Sherrouse, 2016; García-Pérez et al., 2018; Karki et al., 2021). These characteristics of *S. aureus* might contribute to the increased number of differentially expressed proteins in the co-incubation with *C. jejuni.*

In the co-incubation assay with *E. faecium*, 405 proteins were differentially expressed and in the assay with *E. faecalis*, 241 proteins were differentially expressed. The ratio of up-expressed and down-expressed proteins also varied specifically.

**Figure 2:**
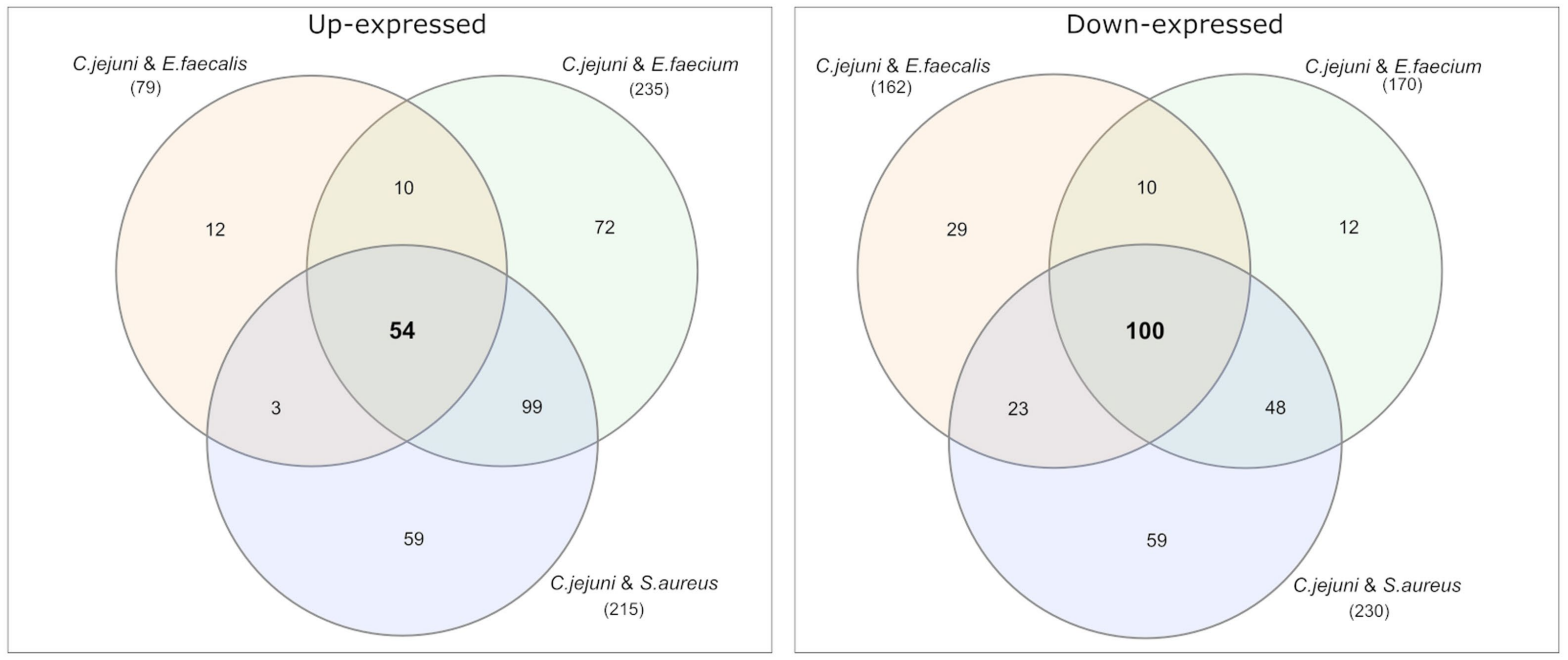
Venn diagrams were used to show the commonly up- and down-expressed proteins in *C. jejuni* during co-incubation with *E. faecalis*, *E. faecium*, and *S. aureus* in the pellet. The analysis revealed that 54 proteins were commonly up-expressed in all three co-incubation approaches, while 100 proteins were commonly down-expressed.

**Figure 3:**
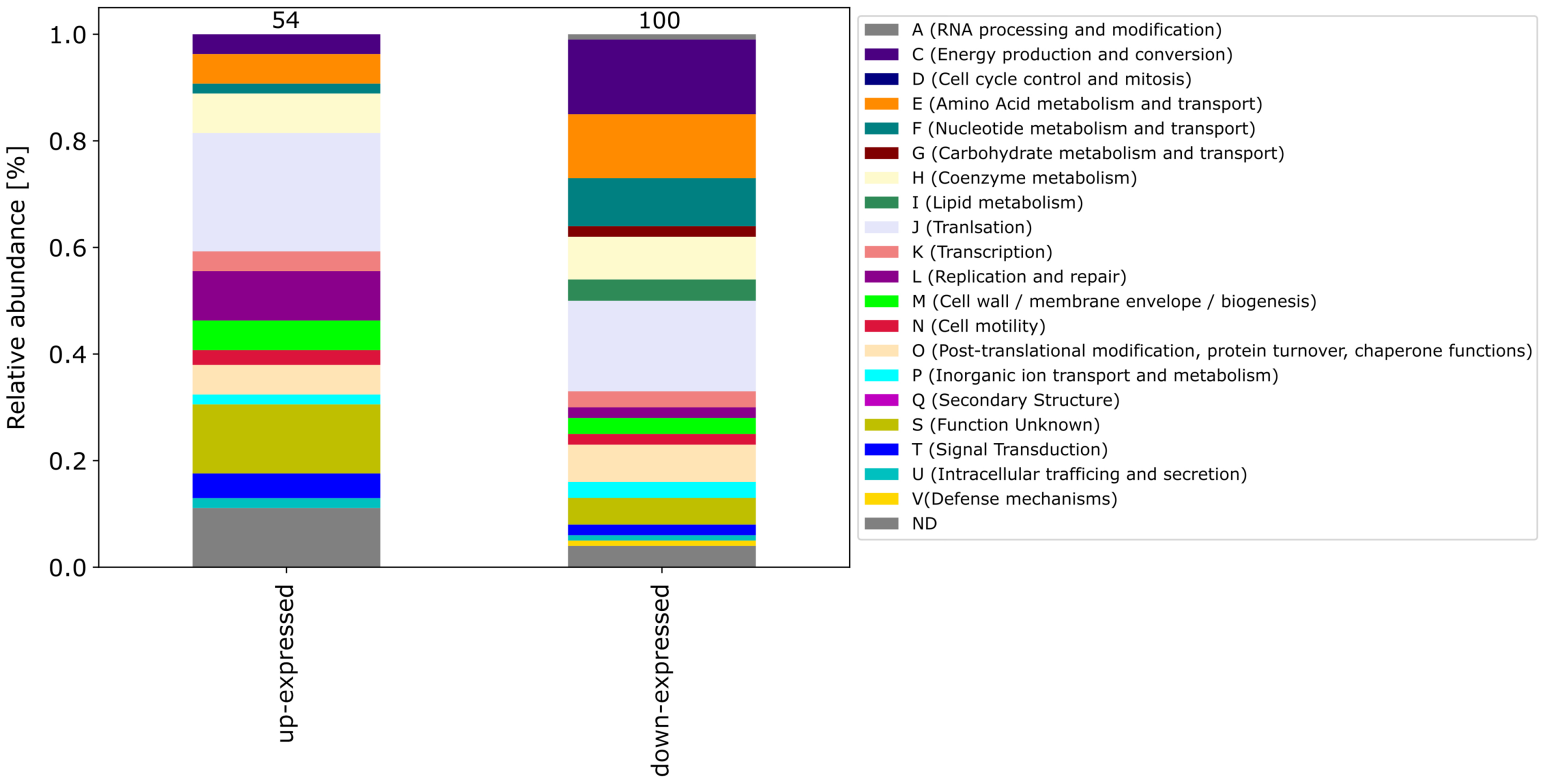
This figure shows the COG-categories of the 54 proteins commonly up-expressed and 100 proteins commonly down-expressed in all three co-incubation approaches. The samples were normalized before analysis. The stacked bar plot displays the percentage distribution of the COG-categories, with different colors representing different categories.

Among the differential expressed proteins, 54 were commonly up-expressed in all three co-incubation approaches and 100 proteins were commonly down-expressed (Figure 2). The distribution of COG-categories differs between commonly up-expressed and down-expressed proteins (Figure 3). Down-expressed proteins are characterized by a higher proportion of the categories C (energy production and conversion), E (amino acid metabolism and transport), F (nucleotide metabolism and transport), I (lipid metabolism) and G (carbohydrate metabolism and transport). In contrast, up-expressed proteins are characterized by a higher proportion of the categories J (translation), L (replication and repair), M (cell wall / membrane / envelope biogenesis) and T (signal transduction).

The differentially expressed proteins in all approaches were sorted according to their difference expression level. We compared the top 20 up- and down-expressed proteins of each co-incubation proteome (see Supplementary Excel file), in order to identify commonly regulated proteins with a high degree of regulation. Four commonly up-expressed proteins were found in the top 20 up-expressed proteins: hemolysin A (A0A0H3PEK7_CAMJJ), a DNA/RNA non-specific endonuclease (A0A0H3PJE6_CAMJJ), a putative lipoprotein (A0A0H3PA71_CAMJJ), and a putative membrane protein (A0A0H3PDB2_CAMJJ). Hemolysin A is associated with the lysis of cells via disruption of the cell membrane. This phenomenon is particularly observed for red blood cells (erythrocytes), as certain pathogens have the ability to cause lysis of these cells to access an iron source (Skals et al., 2010; Wiles and Mulvey, 2013). Therefore hemolysis is considered as an important virulence factor (Elliott et al., 1998; Kielian et al., 2001; Vandenesch et al., 2012). The putative lipoprotein can be associated with cell–cell contact with host epithelial cells, but probably also with other bacteria (Speare et al., 2022). Putative lipoproteins can be considered as virulence factors, as well. Many outer membrane proteins in pathogenic bacteria are virulence factors that enable or facilitate bacterial attachment to host cell surfaces (Gao et al., 2021). The exact function of the putative membrane protein remains unknown. However, this membrane associated protein might also play a role in intercellular communication. Lipoproteins are a diverse group of membrane proteins that play crucial roles in various biological processes, including cellular physiology, cell division, and virulence. They have significant importance and impact on these phenomena (Kovacs-Simon et al., 2011).

Among the top 20 up-expressed proteins in co-incubation were three membrane-interactive proteins, which might indicate an increased adherence to other bacteria through contact with other bacterial species. Enhanced adherence is only one possible reason for the high expression of membrane associated proteins in co-incubation. Whether these effects are due to co-incubation remains unclear. Our study employed *in vitro* co-incubation experiments to investigate proteomic changes. While these experiments allow for controlled conditions and precise analysis, they may not fully replicate the complex interactions that occur within host environments. A genomic assessment could validate this hypothesis. Nevertheless, it is known that in presence of other commensal microbes, some (pathogenic) bacteria can enhance their virulence (Stacy et al., 2016; Fast et al., 2018).

Moreover, four commonly down-expressed proteins were found in the top 20 down-expressed proteins, namely the translation initiation factor IF-3 (IF3_CAMJJ), a DNA-directed RNA polymerase subunit omega (RPOZ_CAMJJ), an ATP synthase subunit beta (ATPB_CAMJJ) and a 6,7-dimethyl-8-ribityllumazine synthase (RISB_CAMJJ).

Some *Campylobacter* strains possess the capability to employ a type 6 secretion system (T6SS), which can be used for communication with their surrounding environment including other bacteria (Chen et al., 2015; Gallique et al., 2017). However, *C. jejuni* 81–176 does not harbor a type 6 secretion system (Liaw et al., 2019), which implies the utilization of alternative mechanisms for bacterial communication. However, other *C. jejuni* strains, for example the strains *C. jejuni* 488, 43,431 or RC039 utilize a type 6 secretion system (Liaw et al., 2019), indicating that cross-talk via a type 6 secretion system-dependent protein secretion would be possible in some *C. jejuni* strains.

### 3.2. The co-incubation response and the bile acid stress response partly overlap

In order to identify proteins that are specifically regulated during co-incubation, we compared the changes in the proteomic profile after co-incubation with the stress response during incubation with bile acids, which was previously shown to trigger a strong proteomic stress response in *C. jejuni* (Masanta et al., 2019). After 3 h incubation with 0.1% DCA, a substantial proportion of *C. jejuni* proteins were differentially expressed. A total of 526 proteins were identified among the up-expressed proteins, which is ~10-fold more than the 54 up-expressed proteins during co-incubation with Gram-positve bacteria. Likewise, 516 proteins were down-expressed after DCA incubation, which is ~5-fold more than the number during co-incubation with Gram-positve bacteria. This leads to the assumption that the exposure to DCA provokes a significantly more pronounced proteomic response compared to the co-incubation scenarios.

**Figure 4:**
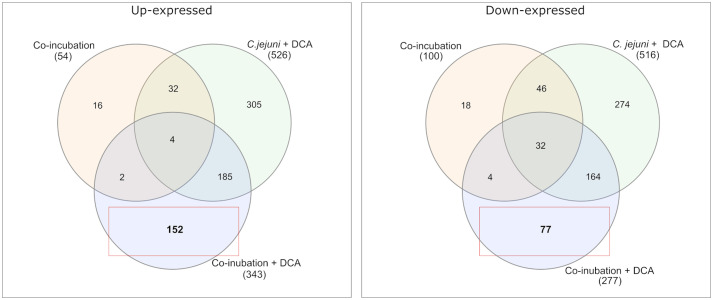
A Venn diagram is presented to compare the commonly up-expressed proteins (left) of *C. jejuni* in co-incubation with *E. faecalis*, *E. faecium*, and *S. aureus* with and without DCA, and the up-expressed proteins of *C. jejuni* with DCA in monoculture. A total of 152 proteins were specifically detected in co-incubation with DCA and not in the other approaches. The down-expressed proteins are shown on the right. The red boxes highlight proteins that are uniquely and specifically expressed in the co-incubation with DCA approach.

**Figure 5:**
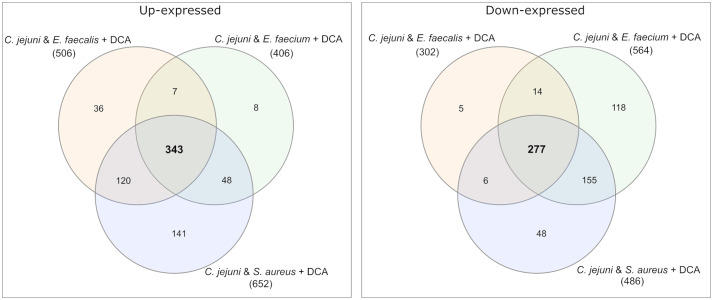
Venn diagrams were used to illustrate the commonly up- and down-expressed proteins of *C. jejuni* in co-incubation with *E. faecalis*, *E. faecium*, and *S. aureus* in the pellet after the addition of DCA. The analysis revealed that 343 proteins were commonly up-expressed in all three co-incubation approaches, while 277 proteins were commonly down-expressed.

Venn diagrams show the overlapping proteins between both approaches (Figure 4 and Supplementary Figures S2, S3). Out of the 54 commonly up-expressed proteins during co-incubation, 36 proteins were also found in *C. jejuni* monoculture with DCA. This indicates that only the 18 remaining proteins are specific for co-incubation (see Supplementary Table S1). Moreover, from the 516 down-expressed proteins in *C. jejuni* in presence of DCA, 78 were shared with the 100 down-expressed proteins in the co-incubation approach (Figure 4), indicating that the 22 remaining proteins are specifically down-expressed in co-incubation (see Supplementary Table S6). In total, 343 proteins were commonly up-expressed and 277 proteins were commonly down-expressed in presence of DCA and co-incubation (Figure 5).

**Figure 6:**
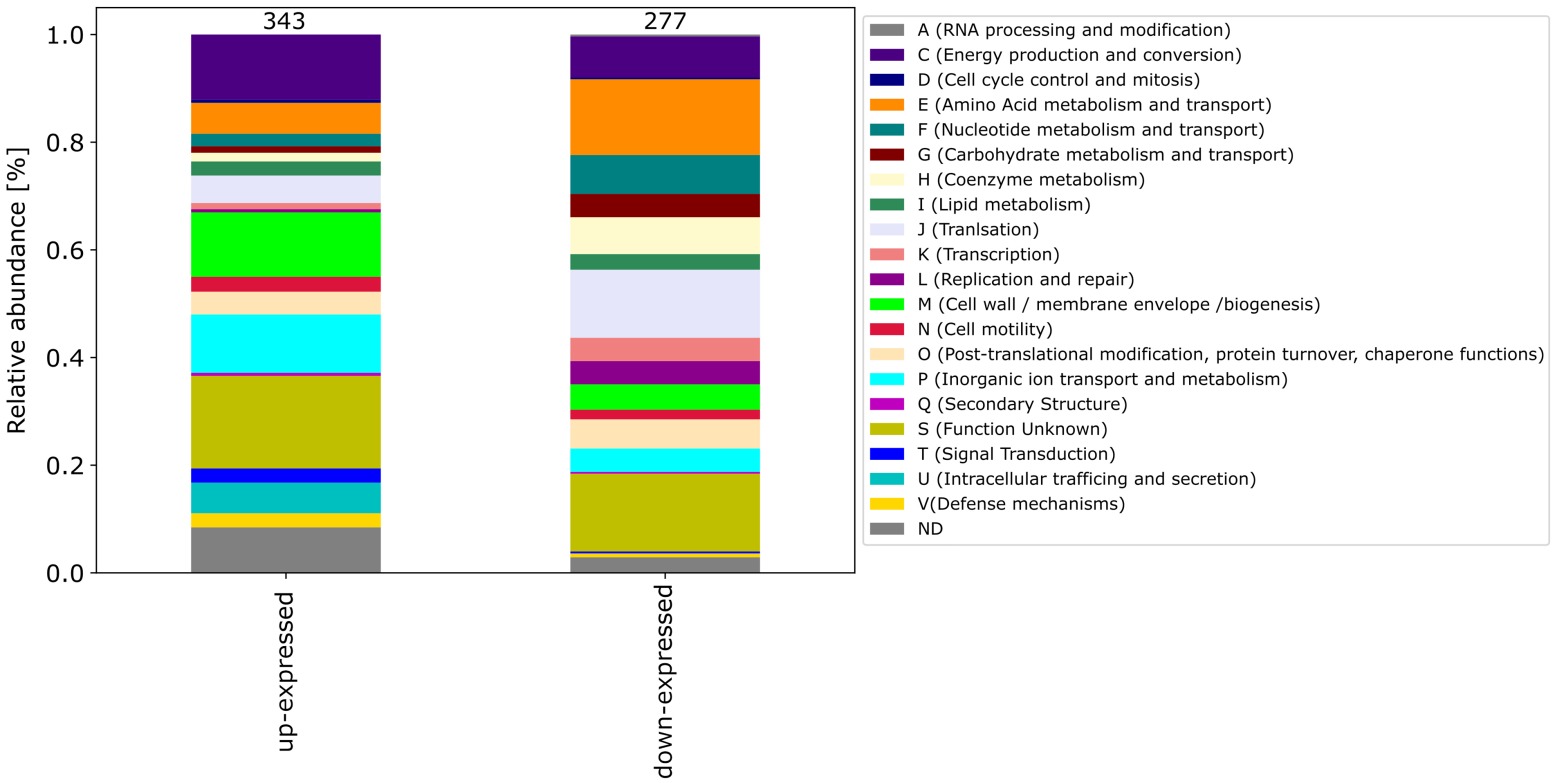
Stacked bar plots were generated to visualize the up- and down-expressed proteins of *C. jejuni* during co-incubation with DCA. A total of 343 up-expressed and 277 down-expressed proteins were assigned to their respective COG categories. The stacked bar plot displays the percentage distribution of COG categories in different colors.

The pattern of the COG categories of differentially proteins in the monoculture approach with DCA differs from commonly expressed proteins in co-incubation (Figures 6^7^). The percentage of up-expressed proteins assigned to the categories J (translation), L (replication and repair) and T (signal transduction) is higher in the co-incubation proteome, while categories C (energy production and conversion), G (carbohydrate metabolism and transport), M (cell wall / membrane / envelope / biogenesis) and V (defense mechanisms) are more present in the monoculture of *C. jejuni* and DCA. Categories C, E, F and J are more down-expressed in the co-incubation approach.

**Figure 7:**
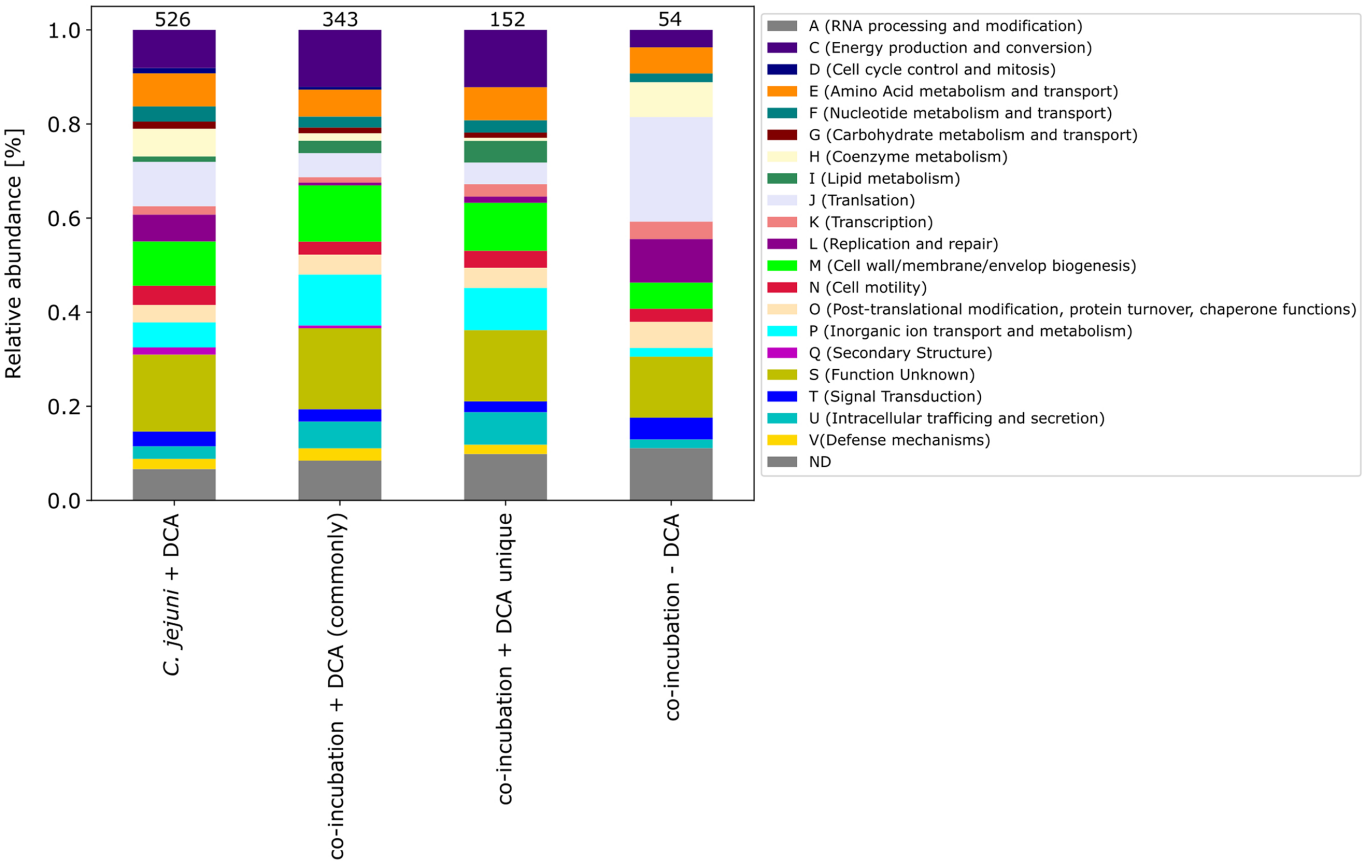
The COG categories of the proteins that were up-expressed in the *C. jejuni* mono-culture approach with DCA, the proteins that were commonly expressed in co-incubation with DCA, and the unique up-expressed proteins of the co-cultivation approach are shown. For comparison, the approach of the 54 up-expressed proteins in co-incubation without DCA is also depicted on the right.

In *C. jejuni* the most relevant mechanism to survive bile acid stress is the CmeABC multidrug efflux pump, that belongs to the resistance nodulation-division (RND) type multidrug efflux systems (Lin et al., 2003). CmeABC consists of a three-gene operon encoding for a membrane fusion protein - CmeA, the efflux pump membrane transporter - CmeB and CmeC, which is the outer membrane lipoprotein (Lin et al., 2002). Knockout mutants of these genes led to significant loss of bile acid resistance (Lin et al., 2003). In a proteomic study, Masanta et al. showed that the proteins belonging to the CmeABC multidrug efflux pump were up-expressed under bile acid stress exposure (Masanta et al., 2019). Furthermore, Malik-Kale et al. found CmeABC and other virulence genes up-regulated under DCA stress in a microarray study (Malik-Kale et al., 2008).

Both publications explored the response of *C. jejuni* to bile acids, but they differ in their methods and focus. Malik-Kale et al. investigated the effect of DCA on the virulence gene expression of *C. jejuni* such as *ciaB, cmeABC*, *dccR*, and *tlyA* using microarray analyses, while Masanta et al. used label-free mass spectrometry to analyze the proteome of *C. jejuni* in response to sublethal concentrations of DCA. However, both studies focused on the response of *C. jejuni* to bile acids and suggest that bile acids can alter the behavior of *C. jejuni*. In contrast to Malik-Kale and colleagues, who focused on the kinetics of cell invasion, Masanta et al. focused on the downregulation of basic biosynthetic pathways and the transcription machinery.

As in Masanta et al.’s study, we used data independent mass spectrometry to obtain the data. However, the focus of our study was the proteome during co-incobation under bile acid stress. Due to the findings in previous work on the reaction of *C. jejuni* to DCA, the presence of CmeA, B or C in all our samples with DCA served as indicator that the proteome under bile acid stress is detected and depicted. In the co-incubation approach without DCA, none of the the CmeABC proteins was detected (Supplementary Table S2).

Among the 22 specifically down-expressed proteins during co-incubation were mostly general metabolic proteins. In the 18 commonly up-expressed proteins during co-incubation, we found proteins that might play a role in the interaction between *C. jejuni* with other bacteria. For example, a conjugative transfer regulon protein (Q9KIR9_CAMJJ) was detected among the up-expressed proteins in all three samples. The presence of this protein indicates that horizontal gene transfer may be occurring between these bacteria, whereby genetic material can be exchanged between different species (Llosa et al., 2002). This mechanism of genetic exchange could allow for the acquisition of novel genetic traits, such as antibiotic resistance or other beneficial genes and indicates a potential for cross-communication between bacteria.

Additionally, the chaperone protein DnaJ was found among these proteins (DNAJ_CAMJJ), indicating an active response toward stress. DnaJ and related Hsp proteins are highly conserved among species and play a role in diverse processes such as folding and unfolding of proteins, translation and ATPase activity of specific chaperones (Qiu et al., 2006). This indicates that the bacteria might be stressed by either the presence of other bacteria or the absence of nutrients.

### 3.3. Co-incubation of *Campylobacter jejuni* with Gram-positive bacteria in the presence of bile acids triggers a unique proteomic response different from the single stimuli

We also studied the proteomic response in the presence of both triggers, DCA plus co-incubation with Gram-positive bacteria. This should reveal the relative influence of the individual triggers on the common response. Among the 18 up-expressed proteins that were specific to co-incubation, only two were up-expressed in the approach of co-incubation with DCA (Figure 4). These proteins were a Histidine kinase (A0A0H3PE96_CAMJJ) and a tRNA modification GTPase MnmE (MNME_CAMJJ) (Supplementary Table S1). Furthermore, of 22 down-expressed proteins that were specific for co-incubation, only four proteins remained down-expressed when DCA was added. The limited number of commonly regulated proteins in co-incubation with and without DCA indicates that DCA seems to suppress the specific co-incubation response to a large extent.

Comparing the co-incubation plus DCA approach to the monoculture of *C. jejuni* with DCA, 185 proteins occurred commonly among the up-expressed candidates, which corresponded to ~37.8% of the 490 proteins that were up-expressed in the monoculture with DCA, excluding the 36 proteins, that also occurred in co-incubation without DCA (Figures 4^5^). This led to the assumption that the additional trigger of co-incubation might also inhibit the expression of a certain amount of the DCA response specific proteins in *C. jejuni.* Moreover, 77 proteins were uniquely down-expressed in the approach of co-incubation plus DCA (Figure 4), while 196 of the 277 down-expressed proteins in this approach were shared with the *C. jejuni* monoculture with DCA.

The proteomes in co-incubation with and without DCA exhibit significant dissimilarities. In total, 152 proteins were found to be specifically up-expressed when both triggers, co-incubation plus DCA, are present. Due to the fact that these 152 proteins occurred only in the approach co-incubation plus DCA, and were not a combination of both triggers, it can be assumed, that the proteomic response in presence of both, DCA and another bacterium possesses a unique character (Figure 4).

Moreover, the respective COG-categories were assigned to these 152 proteins (Figure 7). Compared to the monoculture proteome with DCA, the categories M (cell wall / membrane envelope / biogenesis), P (inorganic ion transport and metabolism) and U (intracellular trafficking) were increased in co-incubation with DCA. A detailed analysis of these 152 proteins revealed a high number of ABC-transporter associated proteins, proteins related to antibiotic resistance, efflux and transport proteins and general membrane proteins (Supplementary Table S5).

**Figure 8:**
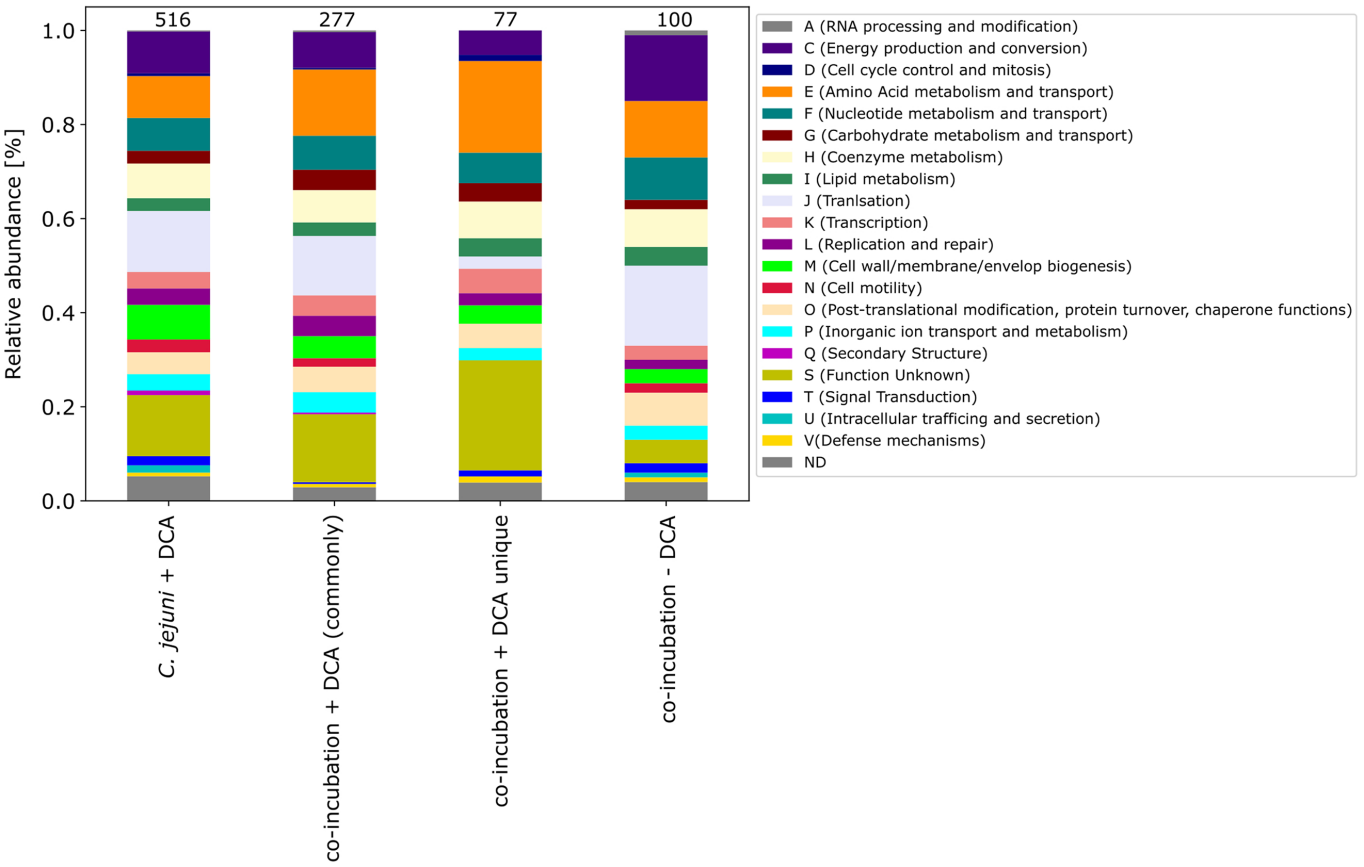
The COG categories of proteins that were down-expressed in the *C. jejuni* mono-culture approach with DCA, as well as the commonly expressed proteins in co-incubation with DCA and the unique up-expressed proteins of the co-cultivation approach are depicted. For comparison, the approach of the 100 down-expressed proteins in co-incubation without DCA is shown on the right.

Furthermore, the COG categories of the 77 proteins commonly exclusively down-expressed in the approach of co-incubation with DCA were determined. When compared to the 516 down-expressed proteins in *C. jejuni* with DCA and the 277 commonly down-expressed proteins in co-incubation with DCA, the pattern of the 77 proteins shows similarities but also differences (Figure 8). An increase of proteins belonging to the category E (amino acid metabolism and transport) was observed and a decrease of proteins belonging to the category J (translation) was observed when compared to the other samples.

### 3.4. Conclusion

In summary, our investigation highlights the proteomic response of *C. jejuni* to co-incubation as well as bile acid stress. We cover a high percentage of the total proteome of *C. jejuni* in our DIA-MS analysis, which demonstrates a small but distinct interaction potential between *C. jejuni* and the other bacteria via membrane-interactive proteins, indicating that the other bacteria contribute to an increased virulence in the environment.

We also report a remarkable overlap between the proteomic response of *C. jejuni* in co-incubation in presence of DCA and the approach of *C. jejuni* monoculture with bile acid.

However, we were able to identify a unique response when both triggers (co-incubation and DCA) are present. This distinct response highlights the complexity of cellular interactions and shows the potential role of *C. jejuni* in proteomic response pathways under these specific conditions and enables future research in the field of proteomic analyses under different influences.

### 3.5. Limitations of the study

One limitation of this study is the labor-intensive nature of DIA MS analysis, which makes it difficult to undertake additional research in this experimental setup. Furthermore, the findings may not be generalizable to other *Campylobacter* strains since only a single strain (*C. jejuni* 81–176) was included. Although the study primarily focused on the *C. jejuni* proteome, there is a lack of comprehensive analysis regarding the proteomic responses of the other bacteria involved in the co-incubation. To provide a more holistic understanding of the interactions and proteomic dynamics within the complex co-incubation system, future research should aim to explore this aspect. We acknowledge that our study primarily focused on proteomic changes in *C. jejuni* during co-incubation with other bacteria. While our results provide valuable insights into proteomic alterations, we did not perform a genomic assessment of *C. jejuni*. Therefore, we are unable to definitively conclude that the observed effects in communication and potential virulence enhancement are solely due to co-incubation.

## Data availability statement

The mass spectrometry proteomics data have been deposited to the ProteomeXchange Consortium via the PRIDE [1] partner repository with the dataset identifier PXD046477.

## Author contributions

CL, WB, UG, and AZ: conceptualization. CL, WB, and AZ: methodology. CL and AD: software. AD, WB, and CL: validation. AD and CL: formal analysis and investigation. AD performed growth curve analysis and prepared bacterial samples. CL performed mass-spectrometric analysis and resources. UG and AZ: data curation. AD and CL: writing—original draft preparation. AD: writing—review and editing. AD, CL, UG, WB, and AZ: visualization. AD prepared all figures. WB and AZ: supervison. AZ: project administration. AZ and UG: funding acqusition. All authors have read and agreed to the published version of the manuscript.
