# Supplementary material for: Characterization of Campylobacter jejuni proteome profiles in co-incubation scenarios
Source: Front Microbiol. 2023 Nov 9;14:1247211. doi: 10.3389/fmicb.2023.1247211 (PMC10666060; doi:10.3389/fmicb.2023.1247211)
Supplement: Supplementary file 1 [file Data_Sheet_1.PDF]

Supplementary Material

Article Title

Characterization of *Campylobacter jejuni* Proteome Profiles in Co-Incubation Scenarios

Authors: Annika Dreyer[1], Christof Lenz[2,3], Uwe Groß[1], Wolfgang Bohne[1,†], Andreas Erich Zautner[1,4,5,†]

[1] Institute for Medical Microbiology and Virology, University Medical Center Göttingen, Göttingen, Germany.

[2] Bioanalytical Mass Spectrometry Group, Max Planck Institute for Multidisciplinary Sciences, Göttingen, Germany.

[3] Department of Clinical Chemistry, University Medical Center Göttingen, Göttingen, Germany.

[4] Institute of Medical Microbiology and Hospital Hygiene, Medical Faculty, Otto-von-Guericke University Magdeburg, Magdeburg, Germany.

[5] Center for Health and Medical Prevention (CHaMP), Otto-von-Guericke University Magdeburg, Magdeburg, Germany.

[†] These authors contributed equally to this work

Supplementary Tables

Suppl. Table 1: 16 *C. jejuni* proteins that were only up-expressed in co-incubation, when compared to co-incubation with DCA and *C. jejuni* monoculture with DCA. Grey marked protein names also occured in co-incubation with DCA.

UniProtKB entry name	Function (presumptive)
A0A0H3P9H2_CAMJJ	HAD-superfamily subfamily IB hydrolase
A0A0H3PBH8_CAMJJ	Tram-like protein
A0A0H3PCM2_CAMJJ	Uncharacterized protein
A0A0H3PE61_CAMJJ	Transcriptional regulator
A0A0H3PGR5_CAMJJ	Dissimilatory sulfite reductase
A0A0H3PGX2_CAMJJ	Uncharacterized protein
A0A0H3PIF4_CAMJJ	HrEpiB
RL20_CAMJJ	50S ribosomal protein L20
YQGF_CAMJJ	Putative pre-16S rRNA nuclease
BPT_CAMJJ	Aspartate/glutamate leucyltransferase
DNAJ_CAMJJ	Chaperone protein DnaJ
MOBA_CAMJJ	Molybdenum cofactor guanylyltransferase
RL18_CAMJJ	50S ribosomal protein L18
Q0Q7I7_CAMJJ	Aminodeoxyfutalosine synthase
Q29W35_CAMJJ	Molybdenum transport system permease
Q9KIR9_CAMJJ	Conjugative transfer regulon protein
MNME_CAMJJ	tRNA modification GTPase MnmE
A0A0H3PE96_CAMJ	Histidine kinase

Suppl. Table 2: 54 commonly up-expressed proteins identified in all three co-cultivation approaches of the pellet. Yellow marked proteins represent candidates that might be involved in conjugation or interaction between the bacteria. Orange marked proteins are related to molybdenum metabolism. Grey marked protein names are proteins that were also found in the approach of *C. jejuni* with DCA in monoculture and are thus not co-cultivation specific.

UniProtKB entry name	Function (presumptive)
A0A0H3P9H2_CAMJJ	HAD-superfamily subfamily IB hydrolase
A0A0H3P9P1_CAMJJ	RNA pseudouridylate synthase
A0A0H3P9Q0_CAMJJ	General secretory pathway protein E
A0A0H3P9S3_CAMJJ	Hydrogenase maturation protease HydD
A0A0H3PA56_CAMJJ	Biotin--acetyl-CoA-carboxylase ligase
A0A0H3PA71_CAMJJ	Lipoprotein, putative
A0A0H3PA79_CAMJJ	DNA ligase, ATP-dependent
A0A0H3PAD8_CAMJJ	MOSC domain protein
A0A0H3PAN2_CAMJJ	LPS glycosyltransferase subfamily
A0A0H3PAN5_CAMJJ	Acetyltransferase
A0A0H3PAU2_CAMJJ	MloB
A0A0H3PAY5_CAMJJ	DNA_rep/recomb_RecO_N domain-containing protein
A0A0H3PBH8_CAMJJ	Tram-like protein
A0A0H3PBW2_CAMJJ	FIST domain-containing protein
A0A0H3PCM2_CAMJJ	Uncharacterized protein
A0A0H3PD15_CAMJJ	Beta-1,4-N-acetylgalactosaminyltransferase
A0A0H3PDB2_CAMJJ	Membrane protein, putative
A0A0H3PDB4_CAMJJ	Uncharacterized protein
A0A0H3PDF2_CAMJJ	DNA-binding response regulator
A0A0H3PDU2_CAMJJ	Peptide ABC transporter, ATP-binding protein
A0A0H3PDV7_CAMJJ	Selenocysteine-specific elongation factor
A0A0H3PE61_CAMJJ	Transcriptional regulator
A0A0H3PE96_CAMJJ	Histidine kinase
A0A0H3PEK7_CAMJJ	Hemolysin A
A0A0H3PF31_CAMJJ	DUF1882 domain-containing protein
A0A0H3PGL6_CAMJJ	Uncharacterized protein
A0A0H3PGN8_CAMJJ	Pseudouridine synthase
A0A0H3PGR5_CAMJJ	Dissimilatory sulfite reductase
A0A0H3PGV5_CAMJJ	Molybdopterin oxidoreductase family protein
A0A0H3PGX2_CAMJJ	Uncharacterized protein
A0A0H3PH28_CAMJJ	Lipooligosaccharide biosynthesis glycosyltransferase
A0A0H3PHP2_CAMJJ	DNA helicase
A0A0H3PIF4_CAMJJ	HrEpiB
A0A0H3PIQ2_CAMJJ	Uncharacterized protein

A0A0H3PJ75_CAMJJ	Uncharacterized protein
A0A0H3PJ87_CAMJJ	TPR domain protein
A0A0H3PJ97_CAMJJ	Bifunctional NAD(P)H-hydrate repair enzyme
A0A0H3PJE6_CAMJJ	DNA/RNA non-specific endonuclease
A0A0H3PJK7_CAMJJ	N-(5'-phosphoribosyl)anthranilate isomerase
RL20_CAMJJ	50S ribosomal protein L20
RS12_CAMJJ	30S ribosomal protein S12
YQGF_CAMJJ	Putative pre-16S rRNA nuclease
RIMM_CAMJJ	Ribosome maturation factor RimM
TRUA_CAMJJ	tRNA pseudouridine synthase A
MNME_CAMJJ	tRNA modification GTPase MnmE
BPT_CAMJJ	Aspartate/glutamate leucyltransferase
UVRC_CAMJJ	UvrABC system protein C
DNAJ_CAMJJ	Chaperone protein DnaJ
MOBA_CAMJJ	Molybdenum cofactor guanylyltransferase
RL18_CAMJJ	50S ribosomal protein L18
Q0Q7I3_CAMJJ	DUF4435 domain-containing protein
Q0Q7I7_CAMJJ	Aminodeoxyfutalosine synthase
Q29W35_CAMJJ	Molybdenum transport system permease
Q9KIR9_CAMJJ	Conjugative transfer regulon protein

Suppl. table 3: The 100 commonly up-expressed proteins identified in all three co-cultivation approaches of the pellet without DCA.

UniProtKB entry name	Function (presumptive)
A0A0H3PAP0_CAMJJ	Thioredoxin
IF3_CAMJJ	Translation initiation factor IF-3
RISB_CAMJJ	6,7-dimethyl-8-ribityllumazine synthase
RPOZ_CAMJJ	DNA-directed RNA polymerase subunit omega
RS15_CAMJJ	30S ribosomal protein S15
A0A0H3PID6_CAMJJ	NADP-dependent malic enzyme, truncation
ATPB_CAMJJ	ATP synthase subunit beta
A0A0H3PF03_CAMJJ	3-oxoacyl-[acyl-carrier-protein] synthase 2
RL6_CAMJJ	50S ribosomal protein L6
A0A0H3P9T1_CAMJJ	Glyceraldehyde-3-phosphate dehydrogenase
A0A0H3P992_CAMJJ	Basal-body rod modification protein FlgD
A0A0H3PHF9_CAMJJ	Uncharacterized protein
A0A0H3P9N6_CAMJJ	LUD_dom domain-containing protein
RL29_CAMJJ	50S ribosomal protein L29
A0A0H3PIW2_CAMJJ	Alkyl hydroperoxide reductase C
PANB_CAMJJ	3-methyl-2-oxobutanoate hydroxymethyltransferase
A0A0H3PAH7_CAMJJ	2-oxoglutarate:acceptor oxidoreductase, alpha subunit
A0A0H3P9E6_CAMJJ	zf-TFIIB domain-containing protein
GLYA_CAMJJ	Serine hydroxymethyltransferase
ATPA_CAMJJ	ATP synthase subunit alpha
CHEY_CAMJJ	Chemotaxis protein CheY homolog
Q2M5Q8_CAMJJ	AccP
A0A0H3PA97_CAMJJ	TolB protein, putative
A0A0H3PBE4_CAMJJ	Chemotaxis protein CheV
A0A0H3PGW3_CAMJJ	Uncharacterized protein
A0A0H3PBJ6_CAMJJ	Uncharacterized protein
A0A0H3PAS8_CAMJJ	Uncharacterized protein
A0A0H3PAX0_CAMJJ	Thiol peroxidase
RL1_CAMJJ	50S ribosomal protein L1
A0A0H3PB76_CAMJJ	Co-chaperone protein DnaJ
RL11_CAMJJ	50S ribosomal protein L11
GLMU_CAMJJ	Bifunctional protein GlmU
A0A0H3PDD9_CAMJJ	Flagellin subunit protein FlaC
RL17_CAMJJ	50S ribosomal protein L17
LUXS_CAMJJ	S-ribosylhomocysteine lyase
A0A0H3PAM0_CAMJJ	Histidine kinase

A0A0H3PAZ0_CAMJJ	Iron ABC transporter, ATP binding subunit
LEU1_CAMJJ	2-isopropylmalate synthase
ATPD_CAMJJ	ATP synthase subunit delta
RL25_CAMJJ	50S ribosomal protein L25
A0A0H3P9M7_CAMJJ	Aconitate hydratase B
CH60_CAMJJ	60 kDa chaperonin
A0A0H3PAP9_CAMJJ	Uncharacterized protein
EFG_CAMJJ	Elongation factor G
DAPA_CAMJJ	4-hydroxy-tetrahydrodipicolinate synthase
SYK_CAMJJ	Lysine--tRNA ligase
ATPE_CAMJJ	ATP synthase epsilon chain
A0A0H3PI81_CAMJJ	Citrate synthase
A0A0H3P9K8_CAMJJ	Iron-sulfur cluster binding protein
Q8GJC6_CAMJJ	Uncharacterized protein
PCKA_CAMJJ	Phosphoenolpyruvate carboxykinase (ATP)
A0A0H3PBN1_CAMJJ	DNA-binding response regulator
A0A0H3PBW9_CAMJJ	ATP-dependent chaperone protein ClpB
A0A0H3PAI9_CAMJJ	UDP-glucose 4-epimerase
A0A0H3PAJ8_CAMJJ	High affinity branched-chain amino acid ABC transporter, ATP-binding protein
A0A0H3PI41_CAMJJ	Uncharacterized protein
A0A0H3P9J6_CAMJJ	Phosphate acetyltransferase
A0A0H3PDJ1_CAMJJ	Oxidoreductase, zinc-binding dehydrogenase family
A0A0H3PAG7_CAMJJ	Purine-binding chemotaxis protein CheW
KDSA_CAMJJ	2-dehydro-3-deoxyphosphooctonate aldolase
A0A0H3PCU1_CAMJJ	Polysaccharide biosynthesis protein
A0A0H3PHE7_CAMJJ	Phospho-2-dehydro-3-deoxyheptonate aldolase
ACKA_CAMJJ	Acetate kinase
A0A0H3P987_CAMJJ	Oxaloacetate decarboxylase, alpha subunit, putative
A0A0H3PHJ5_CAMJJ	DUF3972 domain-containing protein
A0A0H3PJJ8_CAMJJ	Bifunctional chorismate mutase/prephenate dehydratase
TAL_CAMJJ	Transaldolase
A0A0H3PAF7_CAMJJ	Biotin carboxylase
Q0Q7K7_CAMJJ	Chaperone protein DnaK
A0A0H3PHZ1_CAMJJ	Fumarate hydratase class II
A0A0H3PHD6_CAMJJ	Glutamine synthetase
A0A0H3PD61_CAMJJ	Uncharacterized protein
A0A0H3PBL7_CAMJJ	Ferric uptake regulation protein
ACP_CAMJJ	Acyl carrier protein
A0A0H3P9M4_CAMJJ	Aspartate aminotransferase
A0A0H3PCL7_CAMJJ	Ribonucleoside-diphosphate reductase

HIS1_CAMJJ	ATP phosphoribosyltransferase
A0A0H3PB14_CAMJJ	D-isomer specific 2-hydroxyacid dehydrogenase family protein
FOLD_CAMJJ	Bifunctional protein FolD
A0A0H3PEL1_CAMJJ	Methyl-accepting chemotaxis protein
A0A0H3PE25_CAMJJ	ABC transporter, ATP-binding protein
PURA_CAMJJ	Adenylosuccinate synthetase
A0A0H3PB55_CAMJJ	Uncharacterized protein
A0A0H3P999_CAMJJ	HD_domain domain-containing protein
A0A0H3PJ24_CAMJJ	Isocitrate dehydrogenase [NADP]
A0A0H3PA99_CAMJJ	Beta sliding clamp
A0A0H3P9I8_CAMJJ	Arginine decarboxylase
PYRF_CAMJJ	Orotidine 5'-phosphate decarboxylase
A0A0H3P9A5_CAMJJ	Cysteine-rich domain protein
A0A0H3PAU6_CAMJJ	Dihydroorotase
PSEC_CAMJJ	UDP-4-amino-4,6-dideoxy-N-acetyl-beta-L-altrosamine transaminase
A0A0H3P9J5_CAMJJ	Invasion antigen B
A0A0H3P9M5_CAMJJ	Adenylosuccinate lyase
SYE1_CAMJJ	Glutamate--tRNA ligase 1
EFTS_CAMJJ	Elongation factor Ts
A0A0H3PB58_CAMJJ	Aspartokinase
ILVD_CAMJJ	Dihydroxy-acid dehydratase
A0A0H3PBN0_CAMJJ	Carboxypeptidase
A0A0H3PHN4_CAMJJ	Phosphatidylserine decarboxylase
DCUP_CAMJJ	Uroporphyrinogen decarboxylase

Suppl. Table 4: 343 Proteins that were up-expressed in co-cultivation with DCA. Yellow marked proteins represent proteins that are associated with ABC transporters. Orange marked proteins represent efflux proteins. Grey marks represent general membrane proteins. Green marked proteins are known to be involved in antibiotic-resistance.

UniProtKB entry name	Function (presumptive)
RS15_CAMJJ	30S ribosomal protein S15
A0A0H3PH78_CAMJJ	AcrB/AcrD/AcrF family protein
A0A0H3PGP1_CAMJJ	L-lactate permease
A0A0H3PB79_CAMJJ	Efflux pump membrane transporter
A0A0H3PAJ3_CAMJJ	Citrate transporter, authentic frameshift
UPPP_CAMJJ	Undecaprenyl-diphosphatase
A0A0H3PI11_CAMJJ	Uncharacterized protein
A0A0H3PAP3_CAMJJ	Antitoxin
A0A0H3PI52_CAMJJ	50S ribosomal protein L15
RS19_CAMJJ	30S ribosomal protein S19
A0A0H3PEV8_CAMJJ	Penicillin-insensitive transglycosylase
RL24_CAMJJ	50S ribosomal protein L24
A0A0H3PBK7_CAMJJ	Transporter
A0A0H3PCI2_CAMJJ	Uncharacterized protein
A0A0H3PCJ2_CAMJJ	Na+/H+ antiporter family protein
NUOD_CAMJJ	NADH-quinone oxidoreductase subunit D
A0A0H3PEM3_CAMJJ	Integral membrane protein, TerC family
A0A0H3PCE2_CAMJJ	Carbon starvation protein A
A0A0H3PIN6_CAMJJ	Phosphatidylglycerophosphatase A
A0A0H3PA72_CAMJJ	Potassium-transporting ATPase KdpC subunit
Q0Q7I5_CAMJJ	Putative transmembrane transport protein
A0A0H3PJ19_CAMJJ	Uncharacterized protein
RL23_CAMJJ	50S ribosomal protein L23
A0A0H3PCR0_CAMJJ	Cytochrome b
NUOI_CAMJJ	NADH-quinone oxidoreductase subunit I
A0A0H3P9U9_CAMJJ	General glycosylation pathway protein
A0A0H3PAQ1_CAMJJ	Na+/H+ antiporter family protein
A0A0H3PA17_CAMJJ	Sodium/proline symporter
RS10_CAMJJ	30S ribosomal protein S10
A0A0H3PAP7_CAMJJ	Major facilitator family protein
A0A0H3PAF2_CAMJJ	Protein translocase subunit SecD
A0A0H3PI37_CAMJJ	NADH-quinone oxidoreductase
A0A0H3PAN7_CAMJJ	Protein-export membrane protein SecF
A0A0H3PA60_CAMJJ	Anaerobic C4-dicarboxylate transporter

A0A0H3PBB2_CAMJJ	Phospholipase A1
A0A0H3PAC1_CAMJJ	NADH-quinone oxidoreductase, G subunit
A0A0H3PAD4_CAMJJ	Membrane protein, putative
A0A0H3PEA5_CAMJJ	Macrolide-specific efflux protein macA
A0A0H3PGH7_CAMJJ	Sodium transporter, putative
A0A0H3P9R9_CAMJJ	Cytochrome c oxidase, cbb3-type, subunit II
A0A0H3PAT7_CAMJJ	Serine acetyltransferase
A0A0H3PAR9_CAMJJ	Potassium-transporting ATPase potassium-binding subunit
A0A0H3PHD0_CAMJJ	Uncharacterized protein
A0A0H3PAA7_CAMJJ	Cytochrome d ubiquinol oxidase, subunit II
Q0Q7J1_CAMJJ	Putative integral membrane protein
A0A0H3P9Q3_CAMJJ	Translational regulator CsrA
ATPA_CAMJJ	ATP synthase subunit alpha
A0A0H3PCZ1_CAMJJ	Phosphate transporter
A0A0H3PD83_CAMJJ	Signal peptidase I
A0A0H3PE98_CAMJJ	Membrane protein, putative
ATPE_CAMJJ	ATP synthase epsilon chain
A0A0H3PF34_CAMJJ	Flagellar M-ring protein
A0A0H3PA38_CAMJJ	Cytochrome d ubiquinol oxidase, subunit I
A0A0H3P9N4_CAMJJ	Potassium-transporting ATPase ATP-binding subunit
MACB_CAMJJ	Macrolide export ATP-binding/permease protein MacB
A0A0H3PA68_CAMJJ	Cytochrome-c oxidase
A0A0H3P9Y0_CAMJJ	Peptidase, M23/M37 family
A0A0H3PES6_CAMJJ	Uncharacterized protein
A0A0H3PCV7_CAMJJ	Zinc metalloprotease
A0A0H3PAL2_CAMJJ	Lipoprotein, putative
A0A0H3PA10_CAMJJ	Enterochelin ABC transporter, ATP-binding protein
A0A0H3PA29_CAMJJ	Peptidase, M48 family
A0A0H3P9T9_CAMJJ	Alpha-2,3-sialyltransferase
ATPG_CAMJJ	ATP synthase gamma chain
A0A0H3PC37_CAMJJ	Penicillin-binding protein 2
A0A0H3P9Q2_CAMJJ	ammonia-forming);Nitrite reductase (cytochrome
A0A0H3PHI3_CAMJJ	Flagellar biosynthetic protein FliP
A0A0H3PCY2_CAMJJ	Apolipoprotein N-acyltransferase
Q0Q7I6_CAMJJ	Putative serine protease
Q29W27_CAMJJ	Capsular polysaccharide ABC transporter, periplasmic polysaccharide-binding protein
A0A0H3PDA6_CAMJJ	ABC transporter, permease protein
LPXK_CAMJJ	Tetraacyldisaccharide 4'-kinase
A0A0H3PBE5_CAMJJ	Lipoprotein, putative
A0A0H3PA41_CAMJJ	Membrane protein, putative

A0A0H3PGQ9_CAMJJ	Signal peptide peptidase SppA, 36K type
A0A0H3PCE6_CAMJJ	DedA family protein
A0A0H3PA18_CAMJJ	Sodium transporter, putative
RS21_CAMJJ	30S ribosomal protein S21
A0A0H3PCT8_CAMJJ	Cytochrome c biogenesis protein, CcmF/CycK/CcsA family
A0A0H3PEJ9_CAMJJ	Fumarate reductase cytochrome b subunit
A0A0H3PA07_CAMJJ	ABC transporter, permease protein, putative
A0A0H3PJ47_CAMJJ	Outer membrane protein assembly factor BamA
A0A0H3PAW0_CAMJJ	Magnesium transport protein CorA
A0A0H3PBK1_CAMJJ	Sulfatase, putative
Q0Q7J0_CAMJJ	Putative subtilase family serine protease
A0A0H3PBE2_CAMJJ	Uncharacterized protein
A0A0H3P9J7_CAMJJ	ATP synthase F0, B' subunit
A0A0H3PIX5_CAMJJ	Uncharacterized protein
A0A0H3P9K5_CAMJJ	Ser/Thr protein phosphatase family protein
A0A0H3P9D1_CAMJJ	HlyD_D23 domain-containing protein
A0A0H3PAC4_CAMJJ	Cryptic C4-dicarboxylate transporter DcuD, authentic frameshift
A0A0H3PB43_CAMJJ	Outer membrane efflux protein
A0A0H3PDM3_CAMJJ	Serine transporter
LGT_CAMJJ	Phosphatidylglycerol--prolipoprotein diacylglyceryl transferase
A0A0H3PB07_CAMJJ	LPS-assembly protein LptD
A0A0H3PIM6_CAMJJ	Membrane protein, putative
Q9KIS2_CAMJJ	VirB8
A0A0H3PB47_CAMJJ	Membrane protein, putative
A0A0H3PA66_CAMJJ	Anaerobic C4-dicarboxylate transporter
A0A0H3PEF7_CAMJJ	Methyl-accepting chemotaxis protein
A0A0H3P9H8_CAMJJ	2-acylglycerophosphoethanolamine acyltransferase / acyl-acyl carrier protein synthetase
A0A0H3PCS8_CAMJJ	Cation efflux family protein
A0A0H3PBJ3_CAMJJ	Sodium/hydrogen exchanger family protein
A0A0H3PBJ5_CAMJJ	Protein-disulfide reductase
A0A0H3PAQ0_CAMJJ	Lipoprotein, putative
A0A0H3PBF8_CAMJJ	Membrane protein, putative
A0A0H3PAS9_CAMJJ	Sec-independent protein translocase protein TatC
A0A0H3PIQ7_CAMJJ	NADH-quinone oxidoreductase, L subunit
A0A0H3PAC7_CAMJJ	NADH-quinone oxidoreductase, M subunit
A0A0H3PBL0_CAMJJ	Amino acid ABC transporter, permease protein, His/Glu/Gln/Arg/opine family
A0A0H3PAK6_CAMJJ	TonB-dependent heme receptor
A0A0H3PAW6_CAMJJ	Uncharacterized protein
A0A0H3PIG0_CAMJJ	Quinone-reactive Ni/Fe hydrogenase, cytochrome b subunit
A0A0H3PA23_CAMJJ	Lipoprotein, putative

Q8GJB5_CAMJJ	Uncharacterized protein
A0A0H3P9G2_CAMJJ	Cell division protein FtsH, putative
MNTP_CAMJJ	Putative manganese efflux pump MntP
A0A0H3P9W0_CAMJJ	Heavy metal translocating P-type ATPase
A0A0H3PA51_CAMJJ	Ferredoxin-type protein NapG
Q69B93_CAMJJ	Cpp44
A0A0H3PE64_CAMJJ	Membrane protein, putative
A0A0H3PI21_CAMJJ	Cytochrome c-type protein
A0A0H3PA28_CAMJJ	Amino acid ABC transporter, permease protein PEB1
A0A0H3PB91_CAMJJ	Ferredoxin, 4Fe-4S
PLSY_CAMJJ	Glycerol-3-phosphate acyltransferase
A0A0H3P9F9_CAMJJ	Mechanosensitive ion channel family protein
A0A0H3PIB3_CAMJJ	Phosphatidate cytidylyltransferase
A0A0H3P9E2_CAMJJ	Membrane protein, putative
A0A0H3PI86_CAMJJ	Lipoprotein, putative
A0A0H3PIR6_CAMJJ	Peptidase, M23/M37 family
A0A0H3P9J1_CAMJJ	Membrane protein insertase YidC
A0A0H3PER9_CAMJJ	Uncharacterized protein
A0A0H3PBT4_CAMJJ	SPFH domain / Band 7 family protein
SSTT_CAMJJ	Serine/threonine transporter SstT
A0A0H3PBZ7_CAMJJ	4-hydroxybenzoate octaprenyltransferase, putative
A0A0H3P9Q5_CAMJJ	Lipoprotein, putative
A0A0H3PAP6_CAMJJ	Histidine kinase
A0A0H3PAU3_CAMJJ	Uncharacterized protein
A0A0H3PA59_CAMJJ	Radical SAM domain protein
A0A0H3P9F0_CAMJJ	General glycosylation pathway protein
A0A0H3PBD5_CAMJJ	Transporter, putative
A0A0H3PBI9_CAMJJ	Uncharacterized protein
A0A0H3PA77_CAMJJ	Uncharacterized protein
A0A0H3PHV6_CAMJJ	Cell division protein FtsX, putative
ATPB_CAMJJ	ATP synthase subunit beta
A0A0H3PAJ1_CAMJJ	Membrane protein, putative
A0A0H3PAK4_CAMJJ	Amino acid ABC transporter, permease protein, His/Glu/Gln/Arg/opine family
A0A0H3P9B0_CAMJJ	Chromosome partitioning protein, ParA family
A0A0H3PJC1_CAMJJ	Uncharacterized protein
A0A0H3PBE0_CAMJJ	Oligopeptide transporter, OPT family
A0A0H3PCX6_CAMJJ	Uncharacterized protein
A0A0H3PAD7_CAMJJ	RDD domain-containing protein
A0A0H3P9J0_CAMJJ	Amino acid carrier protein
A0A0H3PAJ9_CAMJJ	Flagellar biosynthetic protein FlhB

A0A0H3PA57_CAMJJ	Uncharacterized protein
A0A0H3PJP9_CAMJJ	Protoporphyrinogen IX oxidase
A0A0H3PBJ2_CAMJJ	Alginate O-acetyltransferase AlgI
A0A0H3PCI0_CAMJJ	Disulfide bond formation protein, DsbB family
A0A0H3PB29_CAMJJ	Membrane protein, putative
A0A0H3P9P5_CAMJJ	YihY family protein
A0A0H3PAA5_CAMJJ	Lipoprotein, putative
A0A0H3PAS1_CAMJJ	Uncharacterized protein
A0A0H3PCF2_CAMJJ	Permease, putative
A0A0H3PBI6_CAMJJ	Antibiotic transport protein, putative
A0A0H3PEI3_CAMJJ	Endolytic peptidoglycan transglycosylase RlpA
A0A0H3P9S0_CAMJJ	Copper-translocating P-type ATPase
A0A0H3PIS5_CAMJJ	RND efflux system, membrane fusion protein CmeA
A0A0H3PAX2_CAMJJ	PPK2 domain-containing protein
A0A0H3PA58_CAMJJ	Membrane protein, putative
RISB_CAMJJ	6,7-dimethyl-8-ribityllumazine synthase
A0A0H3PF42_CAMJJ	Penicillin-binding protein
A0A0H3PBC1_CAMJJ	Cation ABC transporter, ATP-binding protein
A0A0H3PD54_CAMJJ	Biotin carboxylase
A0A0H3PAI0_CAMJJ	Uncharacterized protein
A0A0H3PB49_CAMJJ	Methyl-accepting chemotaxis protein
A0A0H3PDD0_CAMJJ	ABC transporter, ATP-binding protein/permease MsbA, putative
LEUD_CAMJJ	3-isopropylmalate dehydratase small subunit
A0A0H3PJB3_CAMJJ	Mechanosensitive ion channel family protein
A0A0H3P9B5_CAMJJ	High affinity branched-chain amino acid ABC transporter, ATP-binding protein
A0A0H3PAU1_CAMJJ	Uncharacterized protein
A0A0H3PB67_CAMJJ	DUF5644 domain-containing protein
Q8GJE7_CAMJJ	VirD4
Q29W29_CAMJJ	Membrane protein, putative
A0A0H3PAE4_CAMJJ	RND efflux system, outer membrane lipoprotein CmeC
A0A0H3PAQ4_CAMJJ	Cyclic peptide ABC transporter, ATP-binding protein/permease
A0A0H3PAX7_CAMJJ	Mur ligase family protein
Q8GJA2_CAMJJ	VirB4
CH60_CAMJJ	60 kDa chaperonin
A0A0H3P996_CAMJJ	Thioredoxin domain-containing protein
A0A0H3PAA3_CAMJJ	Uncharacterized protein
A0A0H3PBU0_CAMJJ	Membrane protein, putative
A0A0H3PIJ8_CAMJJ	Tungsten ABC transporter, ATP-binding protein, putative
A0A0H3PBK2_CAMJJ	Uncharacterized protein
A0A0H3P9B3_CAMJJ	Membrane protein, putative

A0A0H3PJE8_CAMJJ	Membrane protein, putative
A0A0H3PJC9_CAMJJ	Lipoprotein, putative
RL28_CAMJJ	50S ribosomal protein L28
A0A0H3P9D5_CAMJJ	ATP-dependent zinc metalloprotease FtsH
A0A0H3P9C1_CAMJJ	Putative integral membrane protein
A0A0H3PDP3_CAMJJ	Galactosyltransferase
Q2TJD1_CAMJJ	HtrB
A0A0H3P9Z6_CAMJJ	ABC transporter, ATP-binding protein
A0A0H3PIX8_CAMJJ	Cell shape protein MreC
RS16_CAMJJ	30S ribosomal protein S16
A0A0H3PE72_CAMJJ	DNA-binding response regulator, putative
NUOH_CAMJJ	NADH-quinone oxidoreductase subunit H
A0A0H3PH47_CAMJJ	Uncharacterized protein
A0A0H3PBI5_CAMJJ	ABC transporter, periplasmic substrate-binding protein, putative
Q2TJD0_CAMJJ	LgtF
A0A0H3PDK5_CAMJJ	Multidrug resistance efflux transporter, putative
A0A0H3PGI3_CAMJJ	Amino acid ABC transporter, permease protein PEB1
A0A0H3PBV9_CAMJJ	2-oxoglutarate:acceptor oxidoreductase, delta subunit
A0A0H3P9T7_CAMJJ	Methyl-accepting chemotaxis protein
A0A0H3PEG3_CAMJJ	Uncharacterized protein
A0A0H3PAL8_CAMJJ	TrkA domain protein
A0A0H3P9M1_CAMJJ	Chaperone NapD
A0A0H3PIT5_CAMJJ	Na/Pi-cotransporter, putative
A0A0H3P9N8_CAMJJ	TonB system transport protein ExbD, putative
A0A0H3PA33_CAMJJ	3-deoxy-D-manno-octulosonic acid transferase
A0A0H3PB97_CAMJJ	TonB system transport protein ExbB
RL27_CAMJJ	50S ribosomal protein L27
A0A0H3PAE2_CAMJJ	Phosphatidylserine decarboxylase-related protein
A0A0H3PD99_CAMJJ	Uncharacterized protein
RS18_CAMJJ	30S ribosomal protein S18
A0A0H3PBN5_CAMJJ	Membrane protein, putative
A0A0H3PIV2_CAMJJ	Replication-associated recombination protein A
Q0Q7H2_CAMJJ	Protein translocase subunit SecY
A0A0H3PHR9_CAMJJ	Alpha-ketoglutarate permease
A0A0H3PAI3_CAMJJ	Uncharacterized protein
Q9KIS1_CAMJJ	VirB9
A0A0H3P997_CAMJJ	Putative integral membrane protein
A0A0H3P9M8_CAMJJ	Chromosome partitioning protein, ParB family
A0A0H3PDD6_CAMJJ	Histidine kinase
A0A0H3PAF5_CAMJJ	ABC transporter, permease/ATP-binding protein

Q2A945_CAMJJ	Uncharacterized protein
A0A0H3PAH7_CAMJJ	2-oxoglutarate:acceptor oxidoreductase, alpha subunit
A0A0H3PJE1_CAMJJ	Transporter, putative
A0A0H3PAW4_CAMJJ	Transcriptional regulator, putative
A0A0H3PAJ6_CAMJJ	Uncharacterized protein
A0A0H3PAP4_CAMJJ	Oxidoreductase, putative
A0A0H3PA90_CAMJJ	3-octaprenyl-4-hydroxybenzoate carboxy-lyase, putative
A0A0H3PJ52_CAMJJ	SGNH_hydro domain-containing protein
A0A0H3PCK6_CAMJJ	L-asparaginase
A0A0H3P9S9_CAMJJ	Thioredoxin family protein
A0A0H3PH92_CAMJJ	Glycolate oxidase, subunit GlcD
RL21_CAMJJ	50S ribosomal protein L21
A0A0H3P9A7_CAMJJ	Amidohydrolase family protein
A0A0H3PDB9_CAMJJ	Phytase-like domain-containing protein
A0A0H3PED0_CAMJJ	Transcriptional regulator, TetR family
A0A0H3PBM0_CAMJJ	Uncharacterized protein
A0A0H3PBT6_CAMJJ	Ferric uptake regulation protein
A0A0H3P9S4_CAMJJ	DedA family protein
HIS7_CAMJJ	Histidine biosynthesis bifunctional protein HisB
A0A0H3PBB6_CAMJJ	Anthranilate synthase component I
A0A0H3PE85_CAMJJ	DUF4261 domain-containing protein
A0A0H3PHD6_CAMJJ	Glutamine synthetase
A0A0H3P9I4_CAMJJ	Formyltetrahydrofolate deformylase
A0A0H3PEX7_CAMJJ	Uncharacterized protein
ILVC_CAMJJ	Ketol-acid reductoisomerase (NADP(+))
A0A0H3PI91_CAMJJ	Major outer membrane protein
A0A0H3PAD9_CAMJJ	General glycosylation pathway protein
Q0Q7I9_CAMJJ	Glutamine--fructose-6-phosphate aminotransferase [isomerizing]
A0A0H3PBK5_CAMJJ	Phosphoribosylformylglycinamidine synthase subunit PurS
RUVA_CAMJJ	Holliday junction ATP-dependent DNA helicase RuvA
A0A0H3PA02_CAMJJ	Peptidase, M16 family
A0A0H3PBA0_CAMJJ	Carbamoyl-phosphate synthase small chain

Suppl. Table 5: 152 proteins that were exclusively up-expressed in co-cultivation with DCA.

UniProtKB entry name	Function (presumptive)
A0A0H3PH37_CAMJJ	Lipoprotein, putative
A0A0H3PI52_CAMJJ	50S ribosomal protein L15
A0A0H3PEV8_CAMJJ	Penicillin-insensitive transglycosylase
A0A0H3P9C2_CAMJJ	Thioredoxin domain protein
A0A0H3PIN6_CAMJJ	Phosphatidylglycerophosphatase A
A0A0H3PA72_CAMJJ	Potassium-transporting ATPase KdpC subunit
A0A0H3PJ19_CAMJJ	Uncharacterized protein
A0A0H3P9B9_CAMJJ	CjaA protein
A0A0H3P9U9_CAMJJ	General glycosylation pathway protein
A0A0H3P9J8_CAMJJ	CjaC protein
A0A0H3PAF2_CAMJJ	Protein translocase subunit SecD
A0A0H3PAV1_CAMJJ	Chemotaxis protein MotA, putative
A0A0H3P9R9_CAMJJ	Cytochrome c oxidase, cbb3-type, subunit II
A0A0H3PAT7_CAMJJ	Serine acetyltransferase
A0A0H3PIM2_CAMJJ	Enterochelin ABC transporter, periplasmic enterochelin-binding protein, authentic frameshift
A0A0H3PBJ1_CAMJJ	Capsular polysaccharide ABC transporter
Q0Q7J1_CAMJJ	Putative integral membrane protein
A0A0H3PBD1_CAMJJ	Cbb3-type cytochrome c oxidase subunit
A0A0H3PD83_CAMJJ	Signal peptidase I
A0A0H3PJ35_CAMJJ	Cytochrome c family protein
A0A0H3PAL2_CAMJJ	Lipoprotein, putative
A0A0H3PET5_CAMJJ	TonB-dependent receptor, putative, degenerate
A0A0H3PA10_CAMJJ	Enterochelin ABC transporter, ATP-binding protein
A0A0H3PEL1_CAMJJ	Methyl-accepting chemotaxis protein
A0A0H3PBE5_CAMJJ	Lipoprotein, putative
ATPD_CAMJJ	ATP synthase subunit delta
A0A0H3PGL0_CAMJJ	Uncharacterized protein
A0A0H3PBR7_CAMJJ	Uncharacterized protein
A0A0H3PEJ9_CAMJJ	Fumarate reductase cytochrome b subunit
A0A0H3PJ47_CAMJJ	Outer membrane protein assembly factor BamA
A0A0H3PAW0_CAMJJ	Magnesium transport protein CorA
A0A0H3PBD3_CAMJJ	CvpA family protein
A0A0H3PBK1_CAMJJ	Sulfatase, putative
A0A0H3PBE2_CAMJJ	Uncharacterized protein
A0A0H3P9J7_CAMJJ	ATP synthase F0, B' subunit
A0A0H3PIX5_CAMJJ	Uncharacterized protein
A0A0H3PCG1_CAMJJ	DnaJ domain protein

A0A0H3P9K5_CAMJJ	Ser/Thr protein phosphatase family protein
A0A0H3PAC4_CAMJJ	Cryptic C4-dicarboxylate transporter DcuD, authentic frameshift
A0A0H3PDM3_CAMJJ	Serine transporter
LGT_CAMJJ	Phosphatidylglycerol--prolipoprotein diacylglyceryl transferase
A0A0H3PAM3_CAMJJ	ABC transporter, permease protein
A0A0H3P9E8_CAMJJ	Ubiquinol--cytochrome c reductase, cytochrome c1 subunit
A0A0H3PBF8_CAMJJ	Membrane protein, putative
RL29_CAMJJ	50S ribosomal protein L29
A0A0H3PAK6_CAMJJ	TonB-dependent heme receptor
A0A0H3PDZ5_CAMJJ	Proline/betaine transporter, putative, authentic frameshift
Q8GJB5_CAMJJ	Uncharacterized protein
PLSY_CAMJJ	Glycerol-3-phosphate acyltransferase
A0A0H3P9L8_CAMJJ	ATP synthase subunit b
A0A0H3PHN4_CAMJJ	Phosphatidylserine decarboxylase
Q8GJA7_CAMJJ	Uncharacterized protein
A0A0H3PI86_CAMJJ	Lipoprotein, putative
A0A0H3P9J1_CAMJJ	Membrane protein insertase YidC
A0A0H3PER9_CAMJJ	Uncharacterized protein
SSTT_CAMJJ	Serine/threonine transporter SstT
A0A0H3P9Q5_CAMJJ	Lipoprotein, putative
PUR7_CAMJJ	Phosphoribosylaminoimidazole-succinocarboxamide synthase
NUOB_CAMJJ	NADH-quinone oxidoreductase subunit B
A0A0H3PAU3_CAMJJ	Uncharacterized protein
A0A0H3PBI9_CAMJJ	Uncharacterized protein
A0A0H3PAJ9_CAMJJ	Flagellar biosynthetic protein FlhB
A0A0H3PCI0_CAMJJ	Disulfide bond formation protein, DsbB family
A0A0H3PB29_CAMJJ	Membrane protein, putative
A0A0H3PAT4_CAMJJ	ABC transporter, permease protein
A0A0H3PBI6_CAMJJ	Antibiotic transport protein, putative
A0A0H3PAB1_CAMJJ	Membrane protein, putative
A0A0H3P9S0_CAMJJ	Copper-translocating P-type ATPase
A0A0H3PAR7_CAMJJ	Lactamase_B domain-containing protein
A0A0H3PAX2_CAMJJ	PPK2 domain-containing protein
A0A0H3PAE9_CAMJJ	Uncharacterized protein
A0A0H3PA89_CAMJJ	DHOdehase
A0A0H3PBB3_CAMJJ	Ribosome-binding factor A
A0A0H3PBX6_CAMJJ	Chemotaxis protein MotB, putative
A0A0H3P994_CAMJJ	MotA/TolQ/ExbB proton channel family protein
A0A0H3PJB3_CAMJJ	Mechanosensitive ion channel family protein
A0A0H3P9B5_CAMJJ	High affinity branched-chain amino acid ABC transporter, ATP-binding protein

A0A0H3PB67_CAMJJ	DUF5644 domain-containing protein
A0A0H3PAI5_CAMJJ	Lipoprotein, VacJ family
CDTA_CAMJJ	Cytolethal distending toxin subunit A
Q8GJE7_CAMJJ	VirD4
A0A0H3PAE4_CAMJJ	RND efflux system, outer membrane lipoprotein CmeC
A0A0H3PAF6_CAMJJ	Membrane protein, putative
A0A0H3PAS8_CAMJJ	Uncharacterized protein
ACCA_CAMJJ	Acetyl-coenzyme A carboxylase carboxyl transferase subunit alpha
Q8GJA2_CAMJJ	VirB4
A0A0H3P996_CAMJJ	Thioredoxin domain-containing protein
A0A0H3PAA3_CAMJJ	Uncharacterized protein
A0A0H3PGI9_CAMJJ	Uncharacterized protein
A0A0H3PIF6_CAMJJ	Flagellar protein FliL
A0A0H3P9B3_CAMJJ	Membrane protein, putative
A0A0H3PJC9_CAMJJ	Lipoprotein, putative
RL28_CAMJJ	50S ribosomal protein L28
A0A0H3P9D5_CAMJJ	ATP-dependent zinc metalloprotease FtsH
A0A0H3PAJ8_CAMJJ	High affinity branched-chain amino acid ABC transporter, ATP-binding protein
Q2TJD1_CAMJJ	HtrB
A0A0H3P9Z6_CAMJJ	ABC transporter, ATP-binding protein
A0A0H3PIX8_CAMJJ	Cell shape protein MreC
RS16_CAMJJ	30S ribosomal protein S16
A0A0H3PE72_CAMJJ	DNA-binding response regulator, putative
A0A0H3PHB9_CAMJJ	Ubiquinol-cytochrome c reductase iron-sulfur subunit
A0A0H3PGI3_CAMJJ	Amino acid ABC transporter, permease protein PEB1
A0A0H3P9J4_CAMJJ	Arylsulfate sulfotransferase, degenerate
A0A0H3PBV9_CAMJJ	2-oxoglutarate:acceptor oxidoreductase, delta subunit
A0A0H3P9T7_CAMJJ	Methyl-accepting chemotaxis protein
A0A0H3PCL3_CAMJJ	Cytolethal distending toxin, subunit B
A0A0H3PAL8_CAMJJ	TrkA domain protein
A0A0H3P9N8_CAMJJ	TonB system transport protein ExbD, putative
A0A0H3PAV0_CAMJJ	Outer membrane lipoprotein Blc
A0A0H3PB97_CAMJJ	TonB system transport protein ExbB
Q29W36_CAMJJ	Molybdenum ABC transporter, ATP-binding protein
A0A0H3PAE2_CAMJJ	Phosphatidylserine decarboxylase-related protein
A0A0H3PD99_CAMJJ	Uncharacterized protein
A0A0H3PAI8_CAMJJ	MotA_ExbB domain-containing protein
A0A0H3PDB7_CAMJJ	Membrane protein, putative
A0A0H3PHU2_CAMJJ	Mannosyl-glycoprotein endo-beta-N-acetylglucosamidase domain protein
A0A0H3PIV2_CAMJJ	Replication-associated recombination protein A

A0A0H3PHR9_CAMJJ	Alpha-ketoglutarate permease
A0A0H3P9T5_CAMJJ	Iron permease, FTR1 family
PANB_CAMJJ	3-methyl-2-oxobutanoate hydroxymethyltransferase
A0A0H3PA88_CAMJJ	Lipoprotein, putative
A0A0H3P9M8_CAMJJ	Chromosome partitioning protein, ParB family
A0A0H3PB32_CAMJJ	TPR_REGION domain-containing protein
A0A0H3PAH7_CAMJJ	2-oxoglutarate:acceptor oxidoreductase, alpha subunit
A0A0H3PAW4_CAMJJ	Transcriptional regulator, putative
A0A0H3PAJ6_CAMJJ	Uncharacterized protein
A0A0H3P9N6_CAMJJ	LUD_dom domain-containing protein
A0A0H3PB24_CAMJJ	Fibronectin type III domain protein
A0A0H3PBM5_CAMJJ	Uncharacterized protein
A0A0H3PA97_CAMJJ	TolB protein, putative
A0A0H3PCV4_CAMJJ	General glycosylation pathway protein
PEB1C_CAMJJ	Probable ABC transporter ATP-binding protein PEB1C
A0A0H3P9S9_CAMJJ	Thioredoxin family protein
A0A0H3PH92_CAMJJ	Glycolate oxidase, subunit GlcD
RL21_CAMJJ	50S ribosomal protein L21
A0A0H3PHF3_CAMJJ	Peptidyl-prolyl cis-trans isomerase D, homolog
A0A0H3P9A7_CAMJJ	Amidohydrolase family protein
A0A0H3PA44_CAMJJ	Lipoprotein, putative
A0A0H3PE25_CAMJJ	ABC transporter, ATP-binding protein
A0A0H3PED0_CAMJJ	Transcriptional regulator, TetR family
A0A0H3PEZ1_CAMJJ	Fumarate reductase iron-sulfur subunit
A0A0H3PBT6_CAMJJ	Ferric uptake regulation protein
A0A0H3P9S4_CAMJJ	DedA family protein
A0A0H3PH57_CAMJJ	General glycosylation pathway protein
RL11_CAMJJ	50S ribosomal protein L11
TGT_CAMJJ	Queuine tRNA-ribosyltransferase
A0A0H3PAQ2_CAMJJ	Amino acid ABC transporter, ATP-binding protein
A0A0H3PA50_CAMJJ	Lipoprotein, putative
A0A0H3PC19_CAMJJ	SPOR domain-containing protein
A0A0H3P9C5_CAMJJ	Outer membrane liproprotein MapA
AROC_CAMJJ	Chorismate synthase
RUVA_CAMJJ	Holliday junction ATP-dependent DNA helicase RuvA

Suppl. Table 6: The 77 proteins that were exclusively down-expressed in co-cultivation with DCA.

UniProtKB entry name	Function (presumptive)

A0A0H3P9W6_CAMJJ	UPF0033 domain-containing protein
A0A0H3PI25_CAMJJ	Uncharacterized protein
FABH_CAMJJ	3-oxoacyl-[acyl-carrier-protein] synthase 3
A0A0H3PBD8_CAMJJ	Uncharacterized protein
A0A0H3PD65_CAMJJ	High affinity branched-chain amino acid ABC transporter, periplasmic amino acid-binding protein
PAND_CAMJJ	Aspartate 1-decarboxylase
A0A0H3P9L3_CAMJJ	Uncharacterized protein
A0A0H3P9X6_CAMJJ	Nitroreducatase family protein, authentic frameshift
A0A0H3PB83_CAMJJ	Tautomerase
A0A0H3PCQ6_CAMJJ	High affinity branched-chain amino acid ABC transporter, periplasmic amino acid-binding protein
A0A0H3PD80_CAMJJ	Metallo-beta-lactamase family protein
A0A0H3P9B7_CAMJJ	Cytochrome c553
A0A0H3P9H6_CAMJJ	Uncharacterized protein
CLPS_CAMJJ	ATP-dependent Clp protease adapter protein ClpS
PDXJ_CAMJJ	Pyridoxine 5'-phosphate synthase
A0A0H3PIX1_CAMJJ	Uncharacterized protein
Q0Q7I0_CAMJJ	Peptide ABC transporter, periplasmic peptide-binding protein
A0A0H3P9Y1_CAMJJ	Lactamase_B domain-containing protein
A0A0H3PHY2_CAMJJ	Uncharacterized protein
A0A0H3PJ30_CAMJJ	Ribonucleoside-diphosphate reductase subunit beta
A0A0H3PHQ7_CAMJJ	Threonine dehydratase
A0A0H3PI03_CAMJJ	Uncharacterized protein
A0A0H3PAQ5_CAMJJ	Threonine synthase
FABZ_CAMJJ	3-hydroxyacyl-[acyl-carrier-protein] dehydratase FabZ
PXPA_CAMJJ	5-oxoprolinase subunit A
A0A0H3PGM1_CAMJJ	Aspartate ammonia-lyase
NUSB_CAMJJ	Transcription antitermination protein NusB
A0A0H3PBN8_CAMJJ	Beta-lactamase
Q29VW1_CAMJJ	Phosphoheptose isomerase
A0A0H3PBG0_CAMJJ	Thioredoxin-like_fold domain-containing protein
A0A0H3PIA8_CAMJJ	Enoyl-[acyl-carrier-protein] reductase [NADH]
A0A0H3PA70_CAMJJ	Glutamate 5-kinase
A0A0H3PD50_CAMJJ	ADP-L-glycero-D-mannoheptose-6-epimerase
A0A0H3PEI7_CAMJJ	Dihydropteroate synthase
A0A0H3PC06_CAMJJ	Uncharacterized protein
A0A0H3PCS4_CAMJJ	Riboflavin synthase, alpha subunit
A0A0H3PDB4_CAMJJ	Uncharacterized protein
A0A0H3PAJ2_CAMJJ	Cytochrome c family protein
A0A0H3ADZ7_CAMJJ	DNA-binding protein HU

AROQ_CAMJJ	3-dehydroquinate dehydratase
Q939J7_CAMJJ	Flagellin modification protein, PseA
A0A0H3PJ16_CAMJJ	Molybdenum ABC transporter, periplasmic molybdenum-binding protein
A0A0H3PAL5_CAMJJ	Uncharacterized protein
HIS51_CAMJJ	Imidazole glycerol phosphate synthase subunit HisH 1
A0A0H3PIL0_CAMJJ	ATPase_AAA_core domain-containing protein
EFP_CAMJJ	Elongation factor P
A0A0H3PA26_CAMJJ	Peptidyl-arginine deiminase family protein
PYRB_CAMJJ	Aspartate carbamoyltransferase
A0A0H3PAR4_CAMJJ	PolyA polymerase family protein
Q8GJA8_CAMJJ	Uncharacterized protein
Q29W30_CAMJJ	YceI family member
A0A0H3PGJ3_CAMJJ	Acetyltransferase, GNAT family
A0A0H3PAK5_CAMJJ	RNA polymerase sigma factor RpoD
A0A0H3P9P8_CAMJJ	Transketolase
A0A0H3PEK3_CAMJJ	Branched-chain-amino-acid aminotransferase
A0A0H3PIY1_CAMJJ	Uncharacterized protein
HSLV_CAMJJ	ATP-dependent protease subunit HslV
ISPE_CAMJJ	4-diphosphocytidyl-2-C-methyl-D-erythritol kinase
A0A0H3PAR1_CAMJJ	NapL protein
TRUA_CAMJJ	tRNA pseudouridine synthase A
A0A0H3PGX2_CAMJJ	Uncharacterized protein
A0A0H3P991_CAMJJ	Uncharacterized protein
A0A0H3P979_CAMJJ	Hydrolase, carbon-nitrogen family
A0A0H3PD29_CAMJJ	NAD-dependent protein deacylase
A0A0H3P9I3_CAMJJ	Lipoprotein, NLPA family
A0A0H3PJH5_CAMJJ	Carbamoyl-phosphate synthase large chain
A0A0H3PGV5_CAMJJ	Molybdopterin oxidoreductase family protein
A0A0H3PAU4_CAMJJ	Molybdopterin molybdenumtransferase
RPPH_CAMJJ	RNA pyrophosphohydrolase
ALR_CAMJJ	Alanine racemase
Q29VV4_CAMJJ	Thymidine diphospho-4-keto-rhamnose 3,5-epimerase
NADK_CAMJJ	NAD kinase
Q2M5R0_CAMJJ	Uncharacterized protein
A0A0H3PEV5_CAMJJ	Uncharacterized protein
A0A0H3PHX0_CAMJJ	Iron-sulfur cluster-binding domain protein
A0A0H3PBI8_CAMJJ	Phosphoserine phosphatase SerB
A0A0H3PEH2_CAMJJ	Oxidoreductase, Gfo/Idh/MocA family

Suppl. Table 7: 22 down-expressed *C. jejuni* proteins that are only down-expressed in co-incubation, when compared to co-incubation with DCA and C. jejuni monoculture with DCA. Grey marked protein names also occur in co-incubation with DCA.

UniProtKB entry name	Function (presumptive)
RISB_CAMJJ	6,7-dimethyl-8-ribityllumazine synthase
RPOZ_CAMJJ	DNA-directed RNA polymerase subunit omega
RS15_CAMJJ	30S ribosomal protein S15
A0A0H3PID6_CAMJJ	NADP-dependent malic enzyme, truncation
ATPB_CAMJJ	ATP synthase subunit beta
A0A0H3P992_CAMJJ	Basal-body rod modification protein FlgD
A0A0H3PAH7_CAMJJ	2-oxoglutarate:acceptor oxidoreductase, alpha subunit
ATPA_CAMJJ	ATP synthase subunit alpha
Q2M5Q8_CAMJJ	AccP
CH60_CAMJJ	60 kDa chaperonin
ATPE_CAMJJ	ATP synthase epsilon chain
A0A0H3P987_CAMJJ	Oxaloacetate decarboxylase, alpha subunit, putative
A0A0H3PHJ5_CAMJJ	DUF3972 domain-containing protein
A0A0H3PHD6_CAMJJ	Glutamine synthetase
A0A0H3P9M4_CAMJJ	Aspartate aminotransferase
A0A0H3P999_CAMJJ	HD_domain domain-containing protein
A0A0H3P9I8_CAMJJ	Arginine decarboxylase
PSEC_CAMJJ	UDP-4-amino-4,6-dideoxy-N-acetyl-beta-L-altrosamine transaminase
A0A0H3P9T1_CAMJJ	Glyceraldehyde-3-phosphate dehydrogenase
FOLD_CAMJJ	Bifunctional protein FolD
A0A0H3P9M5_CAMJJ	Adenylosuccinate lyase
A0A0H3PBN0_CAMJJ	Carboxypeptidase

Supplementary Figures

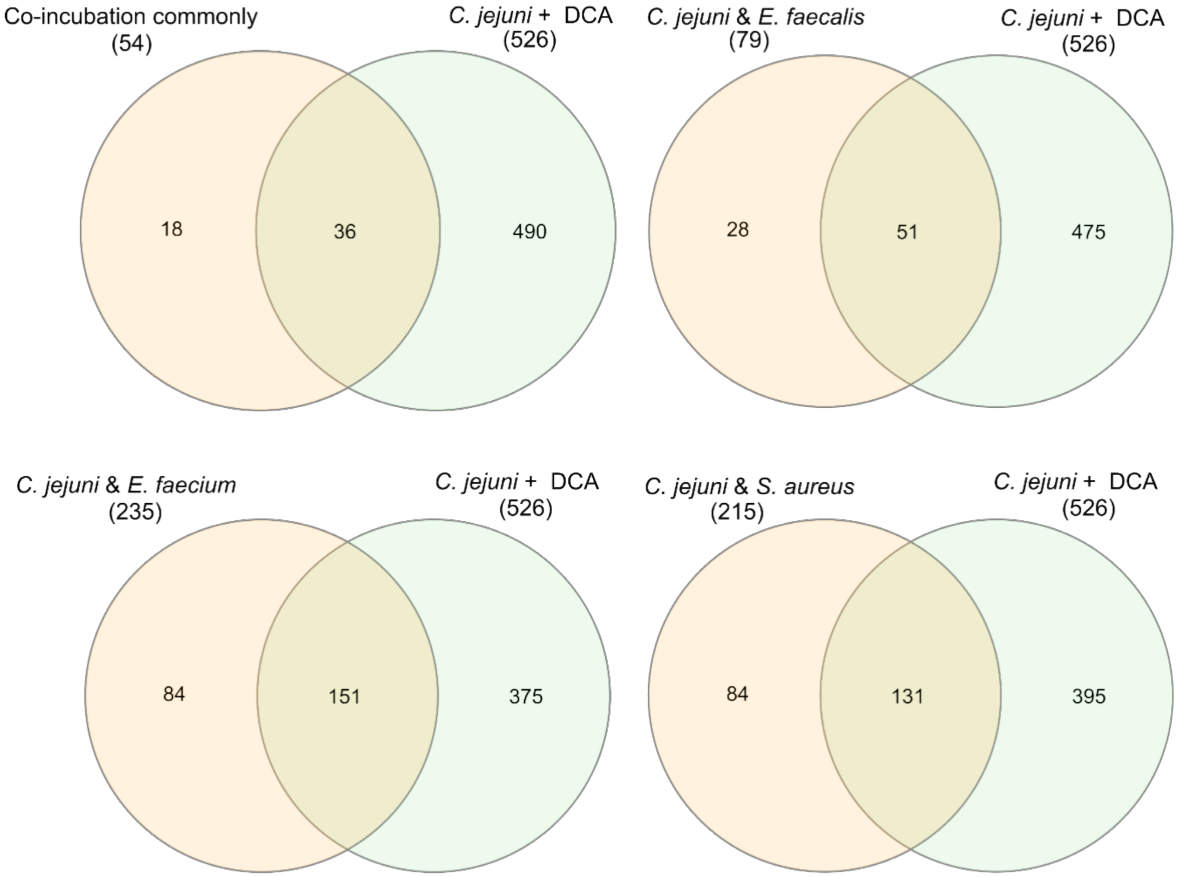

Suppl. Figure 1: Comparison of the up-expressed proteins in *C. jejuni* in presence of DCA compared to each of the co-incubation approaches separately, as well as to the commonly expressed proteins in co-incubation.

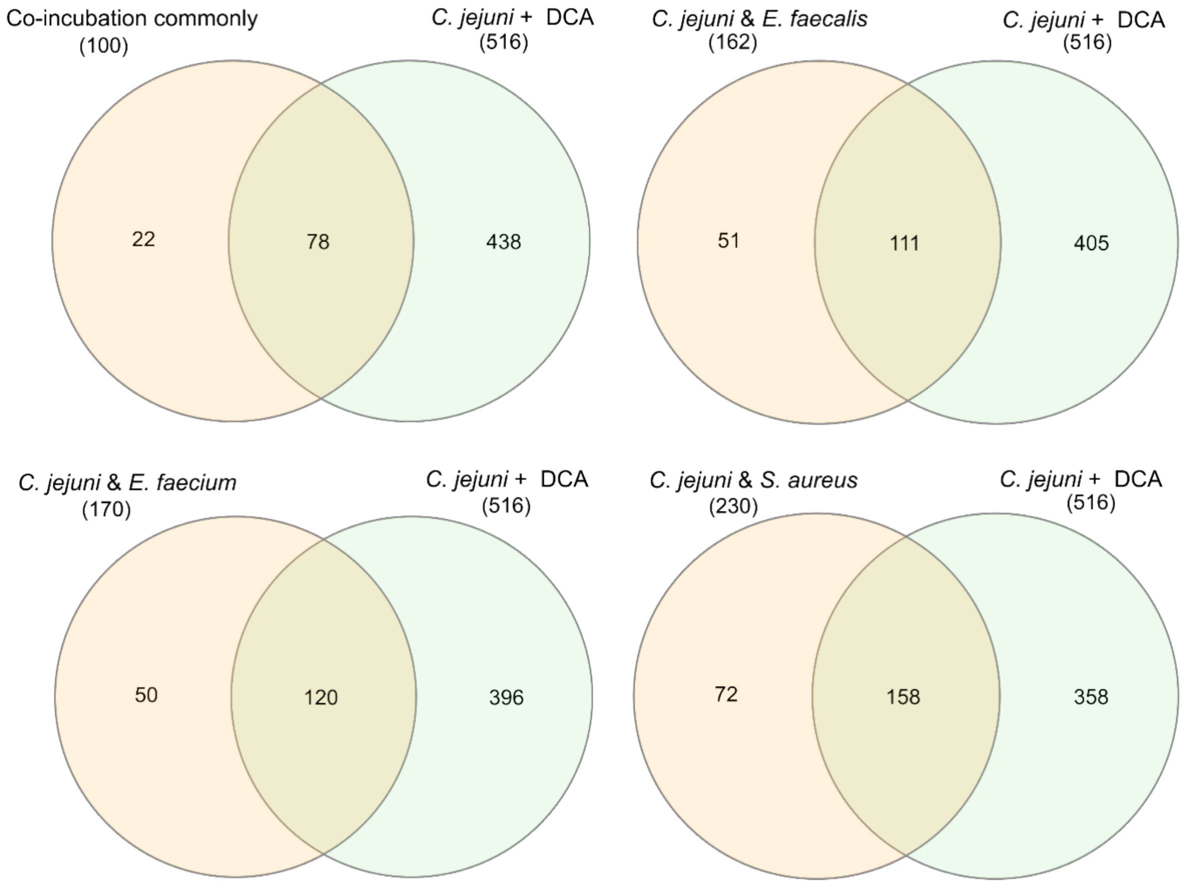

Supp. Figure 2: Comparison of the down-expressed proteins in *C. jejuni* in presence of DCA compared to each of the co-incubation approaches separately, as well as to the commonly expressed proteins in co-incubation.

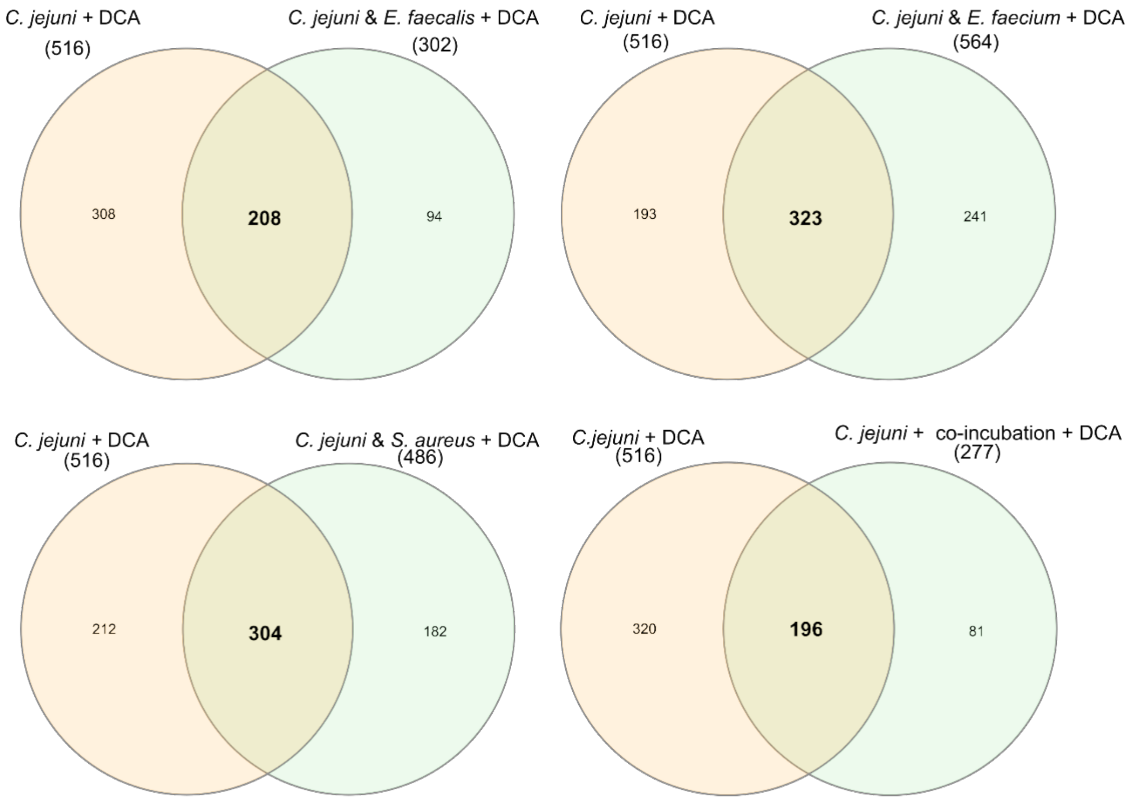

Supp. Figure 3: Comparison of the up-expressed proteins in *C. jejuni* compared to each of the co-incubation approaches separately in presence of DCA, as well as to the commonly expressed proteins in co-incubation and DCA.

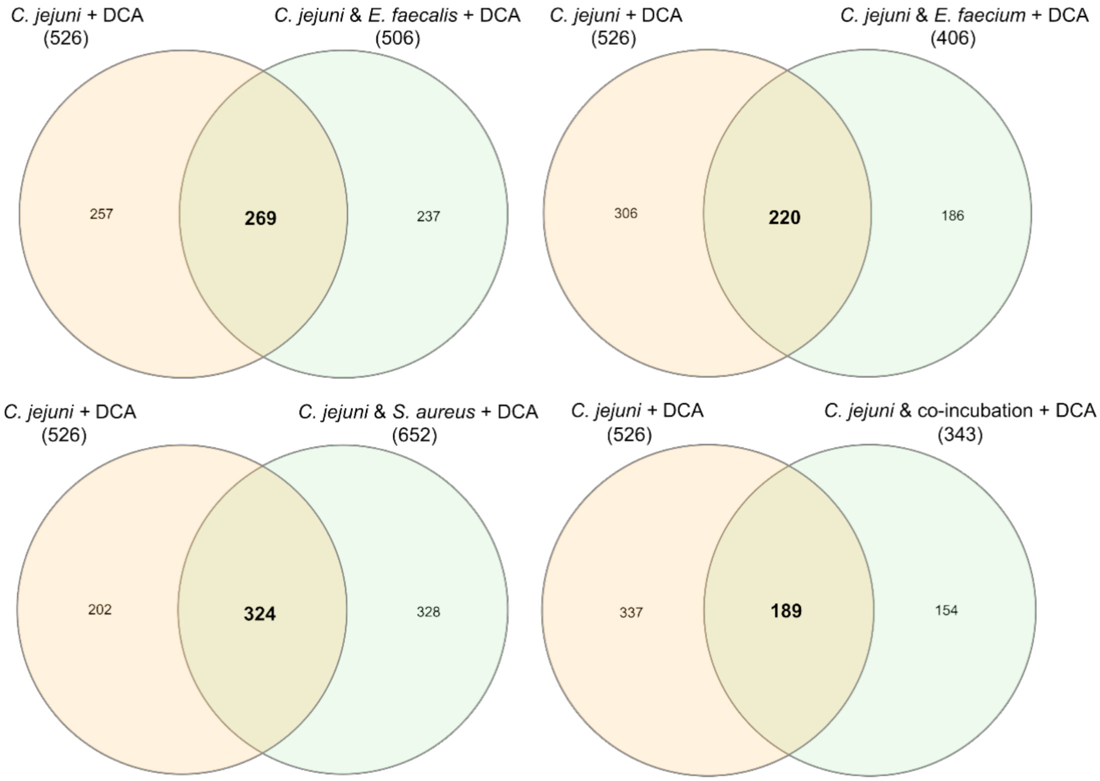

Supp. Figure 4: Comparison of the up-expressed proteins in *C. jejuni* compared to each of the co-incubation approaches separately in presence of DCA, as well as to the commonly expressed proteins in co-incubation and DCA.
